# Bispecific antibodies targeting distinct regions of the spike protein potently neutralize SARS-CoV-2 variants of concern

**DOI:** 10.1126/scitranslmed.abj5413

**Published:** 2021-09-14

**Authors:** Hyeseon Cho, Kristina Kay Gonzales-Wartz, Deli Huang, Meng Yuan, Mary Peterson, Janie Liang, Nathan Beutler, Jonathan L. Torres, Yu Cong, Elena Postnikova, Sandhya Bangaru, Chloe Adrienna Talana, Wei Shi, Eun Sung Yang, Yi Zhang, Kwanyee Leung, Lingshu Wang, Linghang Peng, Jeff Skinner, Shanping Li, Nicholas C. Wu, Hejun Liu, Cherrelle Dacon, Thomas Moyer, Melanie Cohen, Ming Zhao, Frances Eun-Hyung Lee, Rona S. Weinberg, Iyadh Douagi, Robin Gross, Connie Schmaljohn, Amarendra Pegu, John R. Mascola, Michael Holbrook, David Nemazee, Thomas F. Rogers, Andrew B. Ward, Ian A. Wilson, Peter D. Crompton, Joshua Tan

**Affiliations:** 1 Malaria Infection Biology and Immunity Section, Laboratory of Immunogenetics, National Institute of Allergy and Infectious Diseases, National Institutes of Health, Rockville, MD 20852, USA.; 2 Antibody Biology Unit, Laboratory of Immunogenetics, National Institute of Allergy and Infectious Diseases, National Institutes of Health, Rockville, MD 20852, USA.; 3 Department of Immunology and Microbiology, Scripps Research Institute, La Jolla, CA 92037, USA.; 4 Department of Integrative Structural and Computational Biology, Scripps Research Institute, La Jolla, CA 92037, USA.; 5 Integrated Research Facility, Division of Clinical Research, National Institute of Allergy and Infectious Diseases, National Institutes of Health, Frederick, MD 21702, USA.; 6 Vaccine Research Center, National Institute of Allergy and Infectious Diseases, National Institutes of Health, Bethesda, MD 20892, USA.; 7 Flow Cytometry Section, Research Technologies Branch, National Institute of Allergy and Infectious Diseases, National Institutes of Health, Bethesda, MD 20892, USA.; 8 Protein Chemistry Section, Research Technologies Branch, National Institute of Allergy and Infectious Diseases, National Institutes of Health, Rockville, MD 20852, USA.; 9 Division of Pulmonary, Allergy, Critical Care, and Sleep Medicine, Emory University, Atlanta, GA 30322, USA.; 10 New York Blood Center, Lindsley F. Kimball Research Institute, New York, NY 10065, USA.; 11 Division of Infectious Diseases, Department of Medicine, University of California, San Diego, La Jolla, CA 92037, USA.; 12 Skaggs Institute for Chemical Biology, Scripps Research Institute, La Jolla, CA, 92037, USA.

## Abstract

The emergence of severe acute respiratory syndrome coronavirus 2 (SARS-CoV-2) variants of concern threatens the efficacy of existing vaccines and therapeutic antibodies and underscores the need for additional antibody-based tools that potently neutralize variants by targeting multiple sites of the spike protein. We isolated 216 monoclonal antibodies targeting SARS-CoV-2 from plasmablasts and memory B cells collected from patients with coronavirus disease 2019. The three most potent antibodies targeted distinct regions of the receptor binding domain (RBD), and all three neutralized the SARS-CoV-2 Alpha and Beta variants. The crystal structure of the most potent antibody, CV503, revealed that it binds to the ridge region of SARS-CoV-2 RBD, competes with the angiotensin-converting enzyme 2 receptor, and has limited contact with key variant residues K417, E484, and N501. We designed bispecific antibodies by combining nonoverlapping specificities and identified five bispecific antibodies that inhibit SARS-CoV-2 infection at concentrations of less than 1 ng/ml. Through a distinct mode of action, three bispecific antibodies cross-linked adjacent spike proteins using dual N-terminal domain–RBD specificities. One bispecific antibody was greater than 100-fold more potent than a cocktail of its parent monoclonals in vitro and prevented clinical disease in a hamster model at a dose of 2.5 mg/kg. Two bispecific antibodies in our panel comparably neutralized the Alpha, Beta, Gamma, and Delta variants and wild-type virus. Furthermore, a bispecific antibody that neutralized the Beta variant protected hamsters against SARS-CoV-2 expressing the E484K mutation. Thus, bispecific antibodies represent a promising next-generation countermeasure against SARS-CoV-2 variants of concern.

## A bispecific approach to COVID-19

Monoclonal antibodies (mAbs) have become an essential component of coronavirus disease 2019 (COVID-19) treatment. Most mAbs are administered as a cocktail of multiple antibodies that target different regions of the severe acute respiratory syndrome coronavirus 2 (SARS-CoV-2) virion. Here, Cho *et al*. further improved upon this strategy by identifying mAbs that, when combined as bispecific antibodies, neutralized SARS-CoV-2 better than cocktails of the parental mAbs. These bispecific antibodies bound to distinct regions of the SARS-CoV-2 spike protein, neutralized variants of concern, including the Delta variant, and conferred protection when administered to hamsters before SARS-CoV-2 infection. Together, these findings suggest that bispecific antibodies merit further consideration as variant-resistant SARS-CoV-2 therapeutics.

**Figure:**
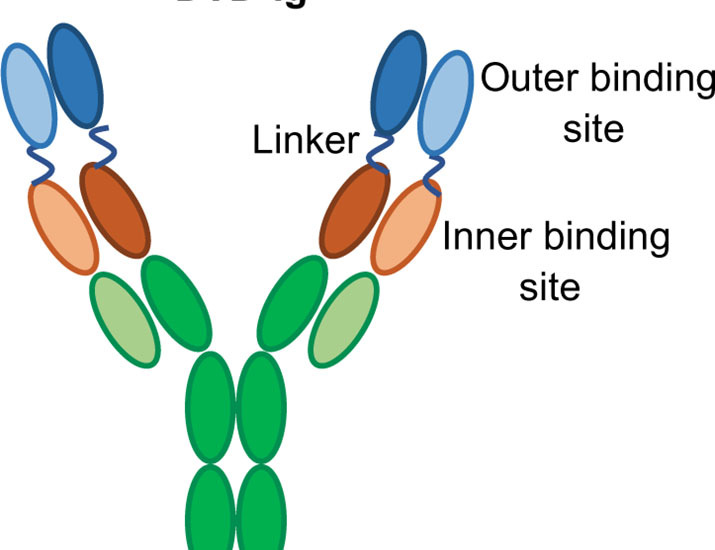

## Teaser

Bispecific antibodies targeting multiple regions of the SARS-CoV-2 spike protein comparably neutralize variants of concern and wild-type virus.

## INTRODUCTION

The severe acute respiratory syndrome coronavirus 2 (SARS-CoV-2) emerged in Wuhan, China in 2019 and rapidly spread across the globe, giving rise to a pandemic that has infected more than 214 million individuals and caused 4.5 million deaths worldwide at the time of writing (*1*). By the end of 2020, several vaccines to prevent SARS-CoV-2 infection became available to the public because of an unprecedented research and production effort. Although more than 5 billion vaccine doses have been administered worldwide to date, the most recent global surge continues to cause more than 600,000 new cases per day of coronavirus disease 2019 (COVID-19), the disease caused by SARS-CoV-2 infection.

In vitro and in vivo experiments, along with observational studies in humans, strongly support a role for SARS-CoV-2–neutralizing antibodies in protection against COVID-19 (*2*–*9*). However, emerging SARS-CoV-2 variants of concern, such as the Alpha (B.1.1.7; first identified in the United Kingdom), Beta (B.1.351; first identified in South Africa), Gamma (P.1; first identified in Brazil), and Delta (B.1.617.2; first identified in India) (*10*–*13*) variants, harbor mutations that may decrease the efficacy of currently available vaccines and therapeutic monoclonal antibodies (mAbs) (*14*–*18*), underscoring the importance of developing new antibody-based tools that potently neutralize variants of concern by targeting diverse sites of the spike protein.

To date, most studies investigating SARS-CoV-2–specific mAbs have used antigen probe–based methods to isolate memory B cells (MBCs) or a mixture of plasmablasts and MBCs (*5*, *7*, *19*–*22*). Here, we used an approach that does not rely on antigen probe–based cell sorting to generate a large panel of mAbs from both plasmablasts and MBCs of recovered patients with COVID-19. We combined potent mAbs with nonoverlapping specificities to generate bispecific antibodies targeting multiple regions of the spike protein that potently neutralize emerging SARS-CoV-2 variants.

## RESULTS

### Characterization of plasma from COVID-19 convalescent donors

**Fig. 1.**
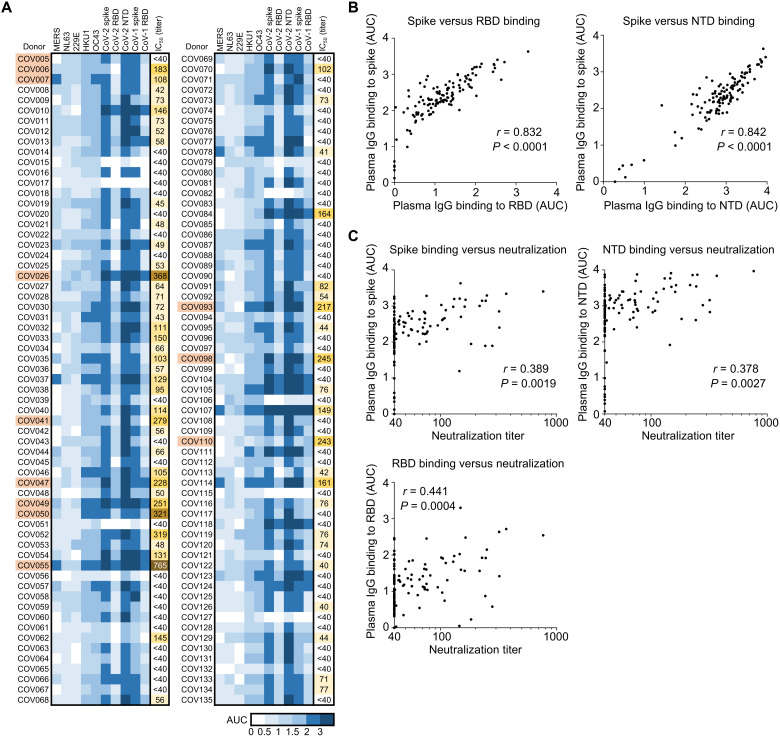
Characterization of plasma from COVID-19 convalescent donors. (**A**) Neutralization titers (IC_50_ values) and values of plasma IgG binding to the spike protein [area under the curve (AUC)] of multiple coronaviruses and specific domains of SARS-CoV-1 and SARS-CoV-2 are shown (*n* = 126 donors). AUC values are shown after subtraction of the negative control antigen. Donors marked in orange were selected for mAb isolation. (**B**) Associations between plasma IgG binding to SARS-CoV-2 spike, RBD, and NTD are shown (*n* = 126 donors). *P* and *r* values were determined by Spearman’s rank correlation. (**C**) The relationship between neutralization and IgG binding to SARS-CoV-2 spike, RBD, and NTD are shown (*n* = 126 donors). *P* and *r* values were determined by Spearman’s rank correlation. Plasma samples that were non-neutralizing at the highest concentration (1:40) are shown on the *y* axis and were excluded from correlation analysis.

To identify the characteristics of circulating antibodies in individuals who successfully controlled SARS-CoV-2 infection, we first examined convalescent plasma samples of 126 individuals in New York City who had recovered from polymerase chain reaction (PCR)–documented SARS-CoV-2 infection. Samples were collected in April 2020 and therefore reflect the B cell response during the first outbreak in the study area. We tested plasma for binding to the spike protein of non–SARS-CoV-2 coronaviruses, as well as to the receptor binding domain (RBD) and N-terminal domain (NTD) of SARS-CoV-2 (Fig. 1A) in a high-throughput bead-based assay analyzed with the IntelliCyt iQue Screener flow cytometer. All donors had detectable antibody binding to at least one non–SARS-CoV-2 spike protein, consistent with previous exposure to seasonal coronaviruses. As expected, most donors also had detectable immunoglobulin G (IgG) antibodies to the SARS-CoV-2 spike protein (119 of 126), RBD (106 of 126), and NTD (122 of 126), and antibody binding to these targets correlated with each other (Fig. 1B). Most donors also had detectable SARS-CoV-2 RBD-specific IgM and IgA (fig. S1A), consistent with the samples being collected from donors in early convalescence. We then tested plasma for neutralization of authentic wild-type SARS-CoV-2 and found a wide range of neutralizing titers from less than 40 to 765 (Fig. 1A). Neutralization potency weakly correlated with amount of IgG specific for SARS-CoV-2 spike, RBD, and NTD, and several plasma samples were non-neutralizing, despite high antibody binding to each of these targets (Fig. 1C), suggesting that the fine specificity of the antibody epitopes is critical for effective neutralization of SARS-CoV-2.

### SARS-CoV-2–specific antibodies derived from plasmablasts and MBCs use diverse V genes and have few mutations

**Fig. 2.**
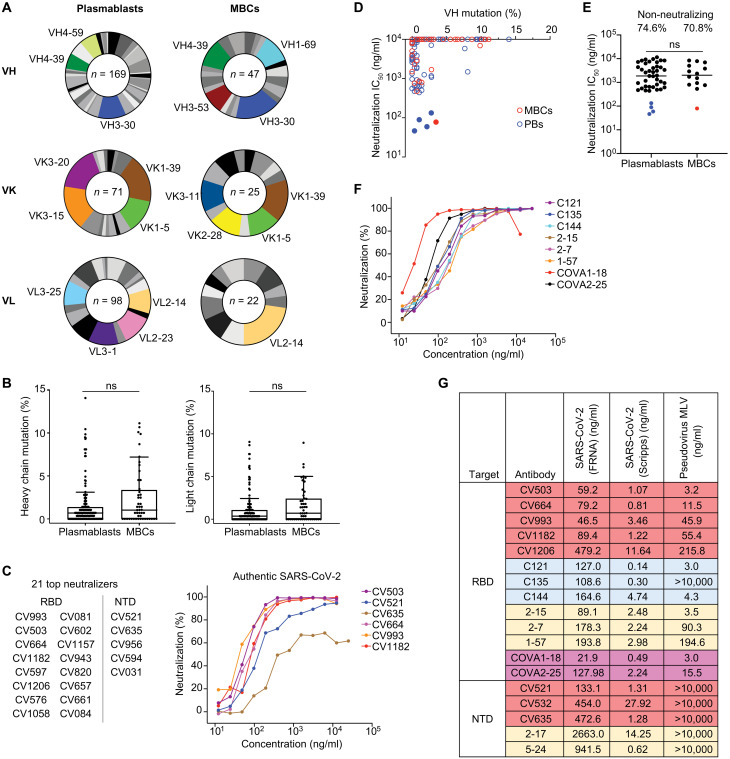
Plasmablasts and MBCs produce potent antibodies against SARS-CoV-2 with diverse V genes and limited mutations. (**A**) VH, VK, and VL gene usage is shown for antibodies isolated from plasmablasts and MBCs. Up to the top four genes in each chart are shown with different colors (genes that were tied for fourth and lower are not highlighted). (**B**) Plots show heavy and light chain gene mutations of antibodies isolated from plasmablasts and MBCs. Percent mutations were compared with the Mann-Whitney *U* test. The middle line shows the median, and the box extends from the first to the third quartile. ns, not significant. (**C**) The top 21 neutralizing antibodies (IC_50_ < 1 μg/ml) are shown by antigen specificity (left), and neutralization curves of selected antibodies are shown (right). (**D**) SARS-CoV-2 neutralization potency is shown plotted against heavy chain mutation frequencies of the antibody panel. The five most potent antibodies are shown as solid circles. (**E**) The neutralization potency of antibodies is shown by cell type. Top values indicate percentages of non-neutralizing antibodies. Horizontal bars indicate median values; data were analyzed using a Mann-Whitney *U* test (non-neutralizing antibodies excluded from calculation). The five most potent antibodies are shown in color. (**F**) SARS-CoV-2 neutralization curves of benchmark antibodies from different groups are shown (*5*, *19*, *20*). (**G**) Neutralization IC_50_ values are shown for antibodies identified in this manuscript and benchmark antibodies in three different neutralization assays. Authentic SARS-CoV-2 FRNA values are from a single experiment done in quadruplicate, authentic SARS-CoV-2 (Scripps) values are an average of two experiments done in duplicate, and pseudovirus (MLV) values are an average of three experiments in duplicate. The colors indicate the different sources of the antibodies: red, this study; blue, (*20*); yellow, (*5*); purple, (*19*).

The specificity and potency of mAbs derived from SARS-CoV-2–specific plasmablasts are poorly characterized (*23*). Therefore, we developed a single-cell assay using the Berkeley Lights Beacon optofluidics device to screen for SARS-CoV-2–specific mAbs secreted by plasmablasts ex vivo and MBCs after in vitro stimulation. Of the 126 donors, we focused on the 9 whose plasma most potently neutralized SARS-CoV-2 in vitro, as well as 3 poor to moderate neutralizers as comparators (Fig. 1A). Circulating plasmablasts (CD19^+^CD27^++^CD38^++^) from these donors were bulk-sorted using fluorescence-activated cell sorting (FACS), distributed individually into nanoliter-volume pens in a microfluidics chip, and then screened directly for secretion of antibodies that bound to beads coated with SARS-CoV-2 spike or RBD (fig. S1B). A total of about 44,000 plasmablasts were screened using this assay, of which 787 supernatants bound to spike or RBD. In parallel, we obtained 291 positive supernatants from MBCs (CD19^+^IgG^+^/IgA^+^) that had been activated in vitro and screened in the same assay. We exported B cells of interest from the microfluidics chip, performed reverse transcription PCR (RT-PCR) to obtain heavy and light chain sequences, and expressed the antibodies recombinantly. In total, we expressed and characterized 169 mAbs targeting SARS-CoV-2 from plasmablasts and 47 from MBCs from the same 12 individuals (fig. S1C and table S1). Of the plasmablast-derived mAbs, 59 targeted the RBD, 64 targeted the NTD, and 46 targeted neither (S2-specific or possibly quaternary), indicating a response to SARS-CoV-2 that is distributed along the entire spike protein. Antibodies isolated from both plasmablasts and MBCs used diverse V genes, with many of the enriched gene families matching those previously reported (*19*, *24*) (Fig. 2A) and partially overlapping between plasmablasts and MBCs. For instance, although genes such as *VH3-30* and *VH4-39* were enriched in both groups, *VH3-53* was more common among MBCs (4th most frequent) than plasmablasts (14th most frequent). We also found that plasmablasts and MBCs had similarly low mutation frequencies (less than 3%) in their heavy and light chain genes (Fig. 2B), consistent with their differentiation from naïve B cells without extensive germinal center experience (*5*, *7*, *19*, *20*, *25*). The markers used for cell sorting did not allow us to distinguish between activated and resting MBCs, but MBC-derived mAbs rarely recognized spike proteins of previously circulating betacoronaviruses, providing further evidence that resting MBCs were not the source of most of the isolated mAbs. A minority of mAbs (about 10%) from plasmablasts and MBCs did have higher mutation frequencies, suggesting that they arose from preexisting MBCs. However, even these antibodies did not cross-react with seasonal coronaviruses (table S1), suggesting the possibility that they may target other pathogens or even self-antigens, as recently described (*26*). To minimize the effects of interdonor variation, we analyzed mAbs isolated from donor COV050, the only individual from whom we obtained similar numbers of plasmablast- and MBC-derived mAbs. We found similar frequencies of heavy chain mutations in antibodies from both cell types (fig. S1D), consistent with the larger unpaired dataset.

### Plasmablasts and MBCs produce highly potent antibodies against SARS-CoV-2

We evaluated the potency of the 216 mAbs in neutralizing authentic SARS-CoV-2 in a high-throughput assay. Most mAbs were non-neutralizing, but several were potent neutralizers with half-maximal inhibitory concentrations (IC_50_) values in the nanogram per milliliter range (Fig. 2C and table S1). Most of the neutralizing antibodies had low mutation frequencies (less than 3%) (Fig. 2D). For antibodies that were originally of the IgA isotype, we compared neutralization of both IgA and IgG forms and found that the IgA generally showed superior neutralization (fig. S1E) (*27*, *28*). Of the 21 antibodies with IC_50_ of less than 1 μg/ml (as IgG), 16 targeted the RBD and 5 bound to the NTD, consistent with previous reports describing the RBD as the primary neutralizing site (*5*, *7*, *22*). All antibodies that did not bind to RBD or NTD did not reach the IC_50_ neutralization threshold of less than 1 μg/ml. Of the 21 most potent mAbs, 16 originated from plasmablasts and 5 from MBCs. The average potency of all neutralizing antibodies from both cell types was similar (Fig. 2E), suggesting that newly differentiated plasmablasts and MBCs can both produce potent antibodies. Potency was also comparable when we only considered antibodies from donor COV050 (fig. S1F).

To determine the relative potency of these mAbs compared to highly potent mAbs described by others (*5*, *19*, *20*), we expressed 10 benchmark IgG1 mAbs: C121, C135, and C144 from Robbiani *et al*. (*20*); COVA1-18 and COVA2-25 from Brouwer *et al*. (*19*); and 2-15, 2-7, 1-57, 2-17, and 5-24 from Liu *et al*. (*5*). To enable an accurate comparison of their relative potency, we performed three different neutralization assays: two with authentic SARS-CoV-2 and one with a SARS-CoV-2 pseudotyped virus (Fig. 2, F and G). The most potent mAbs, particularly CV503 and CV664, performed comparably to the benchmark mAbs across the different assays. Unexpectedly, the NTD-specific antibodies, as well as C135 (*20*), which targets the RBD but does not block angiotensin-converting enzyme 2 (ACE2) binding, did not show efficacy in the pseudovirus assay (Fig. 2G). Furthermore, across the three assays, the relative potency of mAbs was reasonably consistent, but absolute IC_50_ values varied greatly (Fig. 2G), highlighting the importance of using standardized assays to compare antibodies from various sources (see Coronavirus Immunotherapy Consortium; covic.lji.org; and Accelerating COVID-19 Therapeutic Interventions and Vaccines; www.nih.gov/research-training/medical-research-initiatives/activ).

### Potent antibodies against SARS-CoV-2 target diverse epitopes on the RBD and NTD

**Fig. 3.**
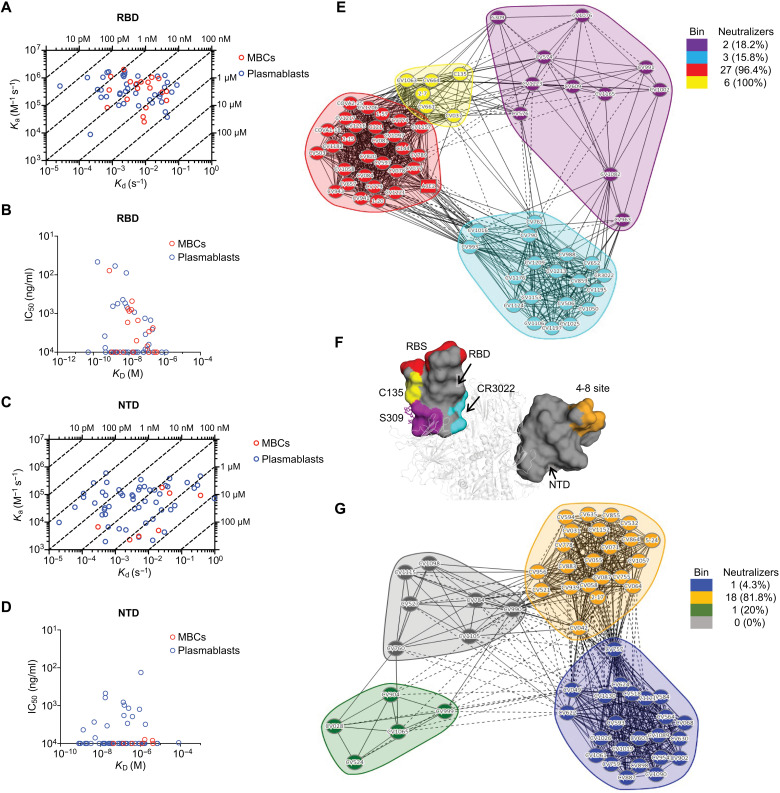
Potent antibodies against SARS-CoV-2 target diverse epitopes on the RBD and NTD. (**A**) An isoaffinity plot of antibodies targeting SARS-CoV-2 RBD is shown (representative of *N* = 2 experiments). The affinity (*K*_D_) values on the top and right of the plot refer to the dashed lines crossing the plot. For example, any antibody falling on the 10 pM dashed line has a *K*_D_ value of 10 pM. (**B**) Neutralization potency is plotted versus affinity of anti-RBD antibodies. (**C**) An isoaffinity plot of antibodies targeting SARS-CoV-2 NTD is shown (representative of *N* = 2 experiments). The affinity (*K*_D_) values on the top and right of the plot refer to the dashed lines crossing the plot. For example, any antibody falling on the 10 pM dashed line has a *K*_D_ value of 10 pM. (**D**) Neutralization potency is plotted versus affinity of anti-NTD antibodies. (**E**) Epitope binning of anti-RBD antibodies is shown (representative of *N* = 2 experiments). ACE2 was only used as an analyte (competitor) and not as a ligand, and all other antibodies were tested as both ligands and analytes. Solid lines indicate two-way competition, and dashed lines indicate one-way competition. The number and percentage of neutralizing antibodies (IC_50_ < 10 μg/ml) in each bin are shown. (**F**) Epitope bins represented by C135 (yellow bin), S309 (purple bin), ACE2 (red bin), CR3022 (cyan bin), as well as the NTD-specific antibody 4-8 (orange) were modeled onto a SARS-CoV-2 spike protein (white cartoon). Antibody 4-8 was not binned successfully in our experiments but binds to a similar region to 2-17 and 5-24 (*5*), which were binned. The epitope sites are color-coded the same as in (E) and (G). N-glycans at the N343 glycan site are represented by sticks. (**G**) Epitope binning of anti-NTD antibodies is shown (representative of *N* = 2 experiments). All antibodies were tested as both ligands and analytes. Solid lines indicate two-way competition, and dashed lines indicate one-way competition. The number and percentage of neutralizing antibodies (IC_50_ < 10 μg/ml) in each bin are shown.

Next, we used high-throughput surface plasmon resonance (SPR) to determine the affinity of the RBD- and NTD-specific mAbs. Overall, RBD-specific mAbs had higher affinity than NTD-specific mAbs (fig. S2A). When stratified by antigen specificity and cell type, RBD-specific mAbs derived from plasmablasts and MBCs had similar affinities, with a few antibodies reaching subnanomolar affinity (Fig. 3, A and B, and fig. S2B). The top four neutralizing mAbs had high affinity (below 10 nM) to the RBD, but other high-affinity antibodies, including the antibody with the highest affinity in our panel, were non-neutralizing (Fig. 3B). In contrast, NTD-specific mAbs from plasmablasts generally had lower affinities than those from MBCs (Fig. 3, C and D, and fig. S2C). Unexpectedly, the most potent mAb had relatively low affinity for the NTD, approaching micromolar dissociation constant (*K*_D_) (Fig. 3D). Analysis of antibodies from donor COV050 showed that plasmablast-derived mAbs had higher affinity than MBC-derived mAbs (fig. S2D), which is consistent with previous work suggesting that higher B cell receptor (BCR) affinity is associated with differentiation into plasma cells (*29*–*31*).

We used high-throughput SPR to perform epitope binning of the antibodies and included several antibodies with known binding sites as controls. The RBD-specific antibodies fell broadly into four bins (Fig. 3, E and F). Most RBD-specific neutralizing antibodies mapped to a bin with ACE2 or a second site containing benchmark antibody C135, which is located on the side of the RBD (Fig. 3F). In contrast, nearly all neutralizing NTD-specific antibodies, including all benchmark NTD-binding antibodies tested (*5*), mapped to a single bin (Fig. 3, F and G), consistent with the recent identification of a single antigenic supersite on the NTD (*32*, *33*). The three most potent mAbs—CV503, CV664, and CV993—mapped to separate epitope bins and did not compete among themselves for binding to RBD. CV503 (red bin) bound to the ACE2 receptor-binding site (RBS), whereas CV664 (yellow bin) and CV993 (cyan bin) targeted opposing sides of the RBD away from the RBS (Fig. 3F). This finding suggested that some of these antibodies may function against the SARS-CoV-2 Alpha and Beta variants, because antibodies that do not directly target the RBS may be more resistant to variant mutations (*16*).

**Fig. 4.**
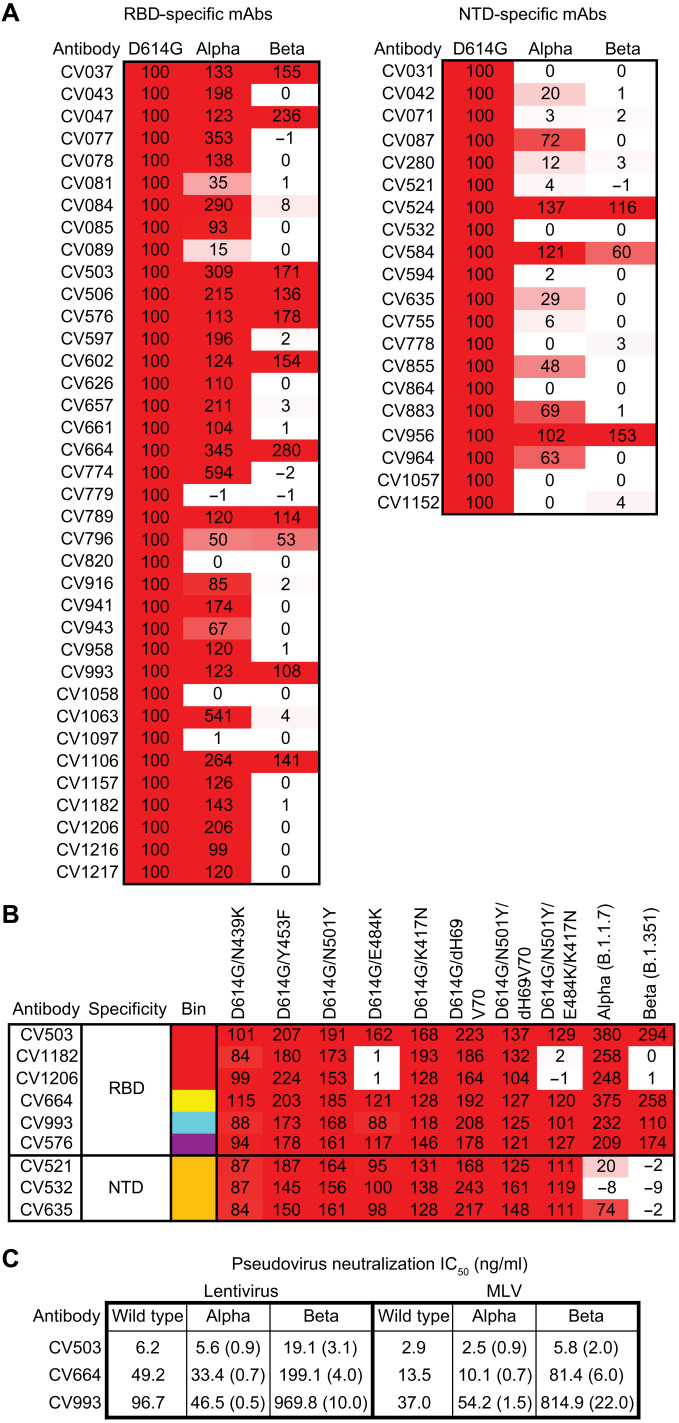
The most potent mAbs identified bind and neutralize SARS-CoV-2 variants of concern. (**A**) Binding of all wild-type SARS-CoV-2–neutralizing antibodies (based on FRNA) to spike protein containing mutations from the Alpha and Beta variants is shown (*N* = 1 experiment). The numbers show the percentages of mAb binding to mutants relative to D614G (which was normalized to 100). (**B**) Binding of mAb panel to spike protein containing mutations from the Alpha and Beta variants is shown (*N* = 1 experiment). The numbers show the percentages of mAb binding to mutants relative to D614G (which was normalized to 100). Colors match the binning shown in Fig. 3. (**C**) Neutralization potency of CV503, CV664, and CV993 against the Alpha and Beta variants relative to wild-type (pseudotyped) SARS-CoV-2 is shown (*N* = 1 experiment). Both lentivirus and MLV pseudoviruses were used. Ratios are shown in parentheses. Numbers smaller than 1 indicate an increase in potency, and numbers larger than 1 indicate a decrease in potency relative to wild type.

We tested the ability of all neutralizing antibodies to bind to Alpha and Beta variant spike protein (Fig. 4A). Twenty-eight of 37 RBD-specific mAbs and 3 of 20 NTD-specific mAbs retained full (greater than 90%) binding to Alpha, but only 10 RBD-specific mAbs and 2 NTD-specific mAbs retained full binding to Beta, consistent with previous findings suggesting that the Beta variant is more successful than the Alpha variant in evading the neutralizing antibody response (*14*, *16*). We narrowed our focus to potent antibodies that targeted distinct epitope bins and screened them against a larger panel of SARS-CoV-2 mutants encoded within the Alpha and Beta variants to enable identification of specific deleterious mutations (Fig. 4B). CV664 and CV993, along with CV576, which mapped to the fourth RBD bin (purple), retained binding to all Alpha and Beta spike protein variants. Unexpectedly, our most potent antibody CV503 (RBS-specific) also retained binding to all variants tested, whereas CV1182 and CV1206, which share a bin with this antibody, failed to bind to the Beta variant because of the E484K mutation. Consistent with these findings, CV503, CV664, and CV993 were able to neutralize the Alpha and Beta variants (Fig. 4C). All three antibodies neutralized the Alpha variant with no loss in potency relative to wild-type virus. CV503 neutralized the Beta variant with a slight (two- to threefold) loss in potency, whereas CV664 and CV993 had a 4- to 22-fold reduction in potency against this variant. In contrast, all three NTD-specific antibodies failed to bind to the Beta spike, and two of three also had sharply reduced or abolished binding to the Alpha spike (Fig. 4B), consistent with previous work suggesting that antibodies targeting the NTD supersite are susceptible to variant mutations (*16*).

### Crystal structure of CV503 reveals a binding site that overlaps with ACE2 with limited interactions with key mutant residues

**Fig. 5.**
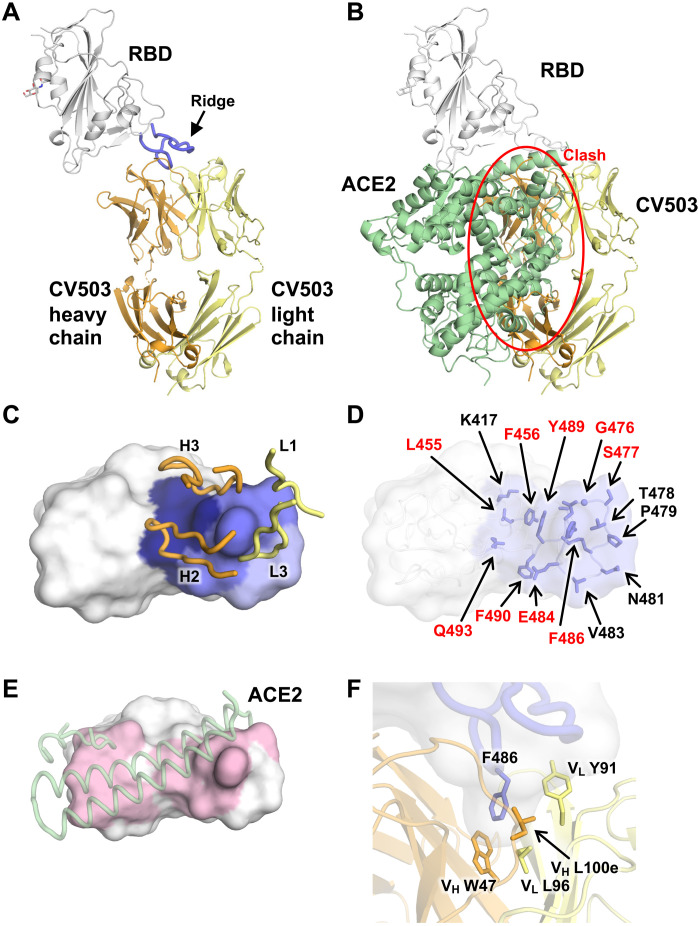
Crystal structure of SARS-CoV-2 RBD in complex with CV503. (**A**) CV503 binds to the ridge region of SARS-CoV-2 RBD. The heavy and light chains of CV503 are shown in orange and yellow, respectively. SARS-CoV-2 RBD is shown in white, where its ridge region (residues 471 to 491) is shown in blue. (**B**) The ACE2/RBD complex structure [PDB ID: 6M0J (*76*)] is superimposed on the CV503/RBD complex. The heavy chain of CV503 (orange) would clash with ACE2 (green) if bound to RBD simultaneously (indicated by red circle). (**C** and **D**) The epitope of CV503 is shown. Epitope residues contacting the heavy chain are in dark blue and light chain in light blue, and residues contacting both heavy and light chains are in dark blue. In (C), complementarity-determining region loops that are directly involved in RBD-binding are labeled. In (D), epitope residues are labeled. Epitope residues that are also involved in ACE2 binding are labeled in red. (**E**) The ACE2-binding site on the RBD is shown in light pink. ACE2 is represented as a semitransparent cartoon in pale green. Epitope residues and ACE2-interacting residues are defined as those with a buried surface area of >0 Å^2^. (**F**) F486 at the ridge region of SARS-CoV-2 RBD (blue) is shown clamped in a hydrophobic pocket formed by the heavy (orange) and light chains (yellow) of CV503.

To understand the mechanism that enabled CV503 to retain binding to the SARS-CoV-2 variants, we determined the crystal structure of Fab CV503 in complex with SARS-CoV-2 RBD at 3.4-Å resolution (Fig. 5 and table S2). Another SARS-CoV-2 targeting Fab, COVA1-16, which binds a different site on the RBD (*19*, *34*), was cocrystallized to enhance crystal lattice formation (fig. S3A). The x-ray structure confirmed that CV503 binds to the RBS of SARS-CoV-2. The buried surface area of SARS-CoV-2 RBD conferred by the heavy and light chains of CV503 is 487 and 290 Å^2^, respectively. CV503 almost exclusively targets the ridge region (residues 471 to 491) of the RBS, with 80% of the antibody interaction focused on this region (Fig. 5A). The ridge region of SARS-CoV-2 RBD dominated the epitope area for several highly potent mAbs in addition to CV503, such as S2E12, where the ridge occupies 80% of the interaction area of the RBD [Protein Data Bank (PDB) ID: 7K45 (*21*)], P17 [79% of area; PDB ID: 7CWO (*35*)], and COVOX-253 [74% of area; PDB ID: 7BEN (*36*)]. These ridge-dominated neutralizing antibodies are encoded by diverse germ lines, including VH1-69, VH3-30, and VH1-58 that encode structurally convergent RBD-targeting antibodies (*36*). This finding highlights the highly immunogenic ridge region as a highly vulnerable area on the SARS-CoV-2 RBD. The structural data indicate that CV503 interferes with the binding of ACE2 to the RBD (Fig. 5, B to E). F486 at the tip of the ridge region is inserted into a hydrophobic pocket formed by the heavy [V_H_ (variable region of immunoglobulin heavy chain) W47 and L100e] and light [V_L_ (variable region of immunoglobulin light chain) L96 and Y91] chains (Fig. 5F). This interaction is similar to those formed by S2E12 (*21*) and COVOX-253 (*36*), which also form hydrophobic pockets with six aromatic amino acids from both heavy and light chains that stack with RBD-F486 (fig. S3, B to I). Similarly, P17 also stacks with RBD-F486 with two tyrosines (*35*). Although both target the same area, S2E12 and COVOX-253 are rotated 50° compared to CV503, whereas P17 is rotated by 85° (fig. S3F). CV503 does not form extensive interactions with K417 and E484, which are located on the periphery of the epitope (Fig. 5D), and does not interact with N501, explaining the resistance of this antibody to mutations in the Alpha and Beta variants.

### Bispecific antibodies potently neutralize SARS-CoV-2

**Fig. 6.**
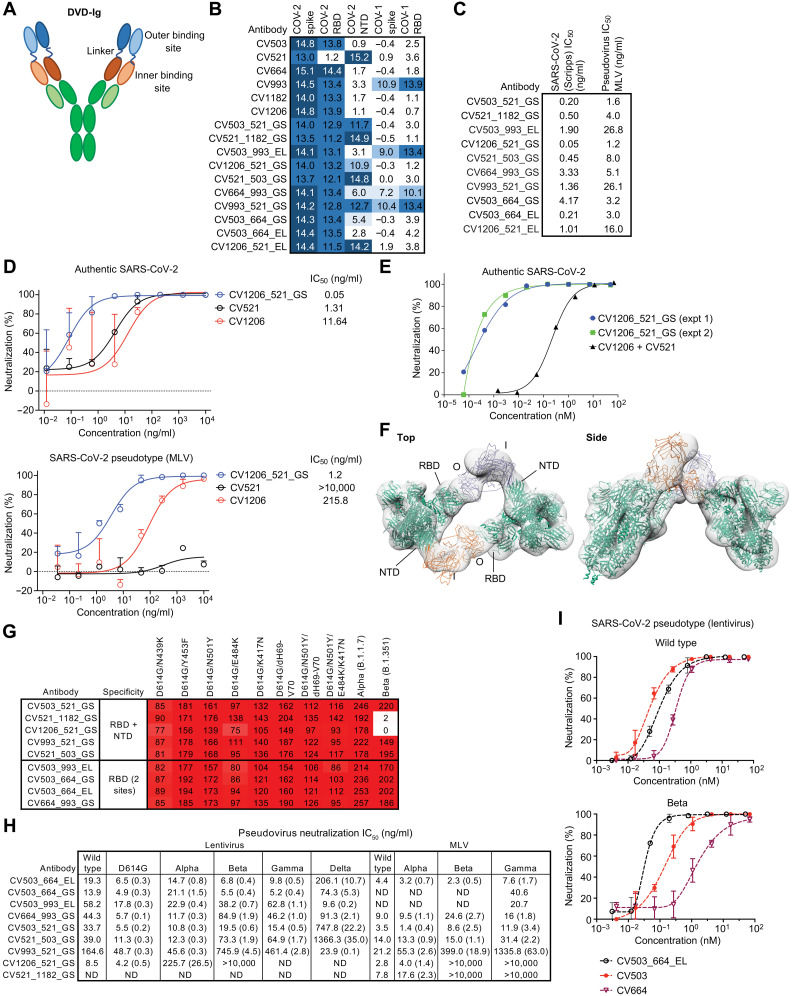
Bispecific antibodies use both binding sites to potently neutralize SARS-CoV-2 and are effective against variants of concern. (**A**) A scheme of DVD-Ig is shown. In our bispecific antibody naming system, the first name refers to the antibody used to make the outer binding site and the second refers to the antibody at the inner binding site. GS or EL refers to the type of linker connecting the two antigen-binding sites. (**B**) Binding of individual and bispecific antibodies to various domains from SARS-CoV-1 and SARS-CoV-2 is shown (representative of *N* = 2 experiments). AUC values are shown after subtraction with the negative control antigen. (**C**) The neutralization potencies of bispecific antibodies against SARS-CoV-2 authentic and pseudotyped virus are shown (MLV). Values are averaged from two experiments done in duplicate. (**D**) Neutralization curves of CV1206_521_GS with SARS-CoV-2 authentic and pseudotyped virus are shown. Curves are from a representative experiment, IC_50_ values for authentic virus are the average from two experiments, and those for the pseudovirus are from an average of two (bispecific) or three (regular antibody) experiments. Points show means ± SD. (**E**) Neutralization potency of CV1206_521_GS versus a cocktail of CV1206 and CV521 is shown, with concentrations shown in the molar scale to enable a fair comparison. For the antibody combination, the values on the *x* axis refers to the concentration of each antibody in the cocktail (10 nM refers to 10 nM CV1206 + 10 nM CV521). expt, experiment. (**F**) A 3D reconstruction of CV1206_521_GS from nsEM images is shown. Two “one RBD up” models (PDB, 6VYB) in green are docked into the reconstruction. Similarly, multiple mock scFvs in orange and purple were docked to approximate the DVD-Ig molecule. O, outer binding site; I, inner binding site. (**G**) Binding of bispecific antibody panel to spike protein containing mutations from the Alpha and Beta variants is shown (*N* = 1 experiment). The numbers show the percentages of mAb binding to mutants relative to D614G (which was normalized to 100). (**H**) Neutralization potencies of bispecific antibodies against D614G, Alpha, Beta, Gamma, and Delta variants relative to wild-type (pseudotyped) SARS-CoV-2 are shown (*N* = 1 experiment). Ratios are shown in parentheses. Numbers smaller than 1 indicate an increase in potency, and numbers larger than 1 indicate a decrease in potency relative to wild type. ND, not determined. (**I**) The potency of CV503_664_EL versus individual component mAbs against wild-type and Beta SARS-CoV-2 pseudotyped virus (lentivirus) is shown. Points show means ± SD.

Given that the most potent mAbs bound to nonoverlapping sites, we assessed possible synergy between these antibodies. We tested RBD-specific antibodies CV503, CV664, CV993, and CV1182, along with the most potent NTD-binder (and fifth best overall neutralizer) CV521 in an initial screen with pairwise combinations between all noncompeting antibodies (fig. S4A). Most combinations, including all RBD-NTD pairs, were additive or inhibitory, but two RBD-specific pairs, CV503 and CV664, as well as CV664 and CV993, showed evidence of synergy at two concentrations. To more rigorously assess synergy, we tested these antibody pairs in neutralization assays in two different laboratories. Follow-up tests with a larger range of concentrations gave inconsistent results, making it unclear whether these antibodies were truly synergistic (fig. S4, B and C). We then hypothesized that merging multiple antibody specificities in the same molecule might provide a different effect than simply mixing two antibodies. Thus, we designed and produced bispecific dual variable domain (DVD)–Ig antibodies combining the variable regions of the potent neutralizers with two types of linkers [GS or EL (*37*)]. This form of a bispecific antibody is relatively easy to express and has a similar structure to standard IgG, except for the addition of an antigen-binding domain on top (outer domain) of the native binding domain (inner domain) through a flexible linker (Fig. 6A). We successfully expressed 10 DVD-Ig antibodies, and SDS–polyacrylamide gel electrophoresis and size exclusion chromatography confirmed that 9 of 10 antibodies contained a single dominant product with the expected molecular weight (fig. S5, A and B). We found that bispecific antibodies that combined an RBD-specific and NTD-specific antibody retained binding to both domains (Fig. 6B). Moreover, SPR experiments confirmed that bispecific antibodies containing two different RBD-binding sites were able to use both sites (fig. S5C). For instance, CV503_664_GS, which combined CV503 and CV664, was able to bind RBD previously attached to either component antibody (fig. S5C). Negative-stain electron microscopy (nsEM) revealed that the 10 bispecific antibodies each cross-linked two to four spike proteins (fig. S6A), and 3 of 10 antibody-spike complexes were amenable to three-dimensional (3D) refinement (fig. S6B). We tested the panel of bispecific antibodies in authentic SARS-CoV-2 and pseudovirus neutralization assays (Fig. 6C). Five bispecific antibodies—CV503_521_GS, CV521_1182_GS, CV1206_521_GS, CV521_503_GS, and CV503_664_EL—were able to neutralize authentic SARS-CoV-2 with an IC_50_ of less than 1 ng/ml (Fig. 6C). CV1206_521_GS was the most potent neutralizing antibody in the panel (including all bispecifics and native monoclonal IgGs), which was unexpected given the lower potency of antibody CV1206 compared to other RBD-specific antibodies (Figs. 2G and 6D). CV1206_521_GS neutralizes SARS-CoV-2 with greater than 100-fold higher potency than a cocktail of its constituent antibodies CV1206 and CV521 (Fig. 6E). We found that CV1206_521_GS uses its inner and outer Fab domains to cross-link NTD and RBD in adjacent spike proteins (Fig. 6F), a mode of action that is unavailable to conventional mAbs, even when used in combination. Together, these data suggest that pairing suitable antibodies in a bispecific format can markedly improve potency by introducing new mechanisms of action.

### Bispecific antibodies are resistant to mutations in emerging SARS-CoV-2 variants

Another potential advantage of bispecific antibodies is resistance to current and future viral escape mutants because these antibodies target multiple sites on the spike protein. We tested the bispecific antibodies for binding to spike proteins carrying individual and total mutations encoded by the Alpha and Beta variants. Only the bispecific antibodies whose two components both lost binding to the SARS-CoV-2 variants, such as CV1206_521_GS, were unable to bind and neutralize these variants (Fig. 6, G and H), suggesting that this antibody form is more resistant to spike mutations than regular mAbs. To confirm the binding results and investigate the full neutralization range of the bispecific antibodies, we tested their ability to neutralize SARS-CoV-2 D614G, Alpha, Beta, Gamma, and Delta pseudotyped virus. All the bispecific antibodies neutralized D614G with no loss of efficacy (Fig. 6H). All dual-RBD binders and most bispecific antibodies that contained CV521 (which had sharply reduced binding to Alpha as a mAb) effectively neutralized the Alpha variant with equal potency compared to wild-type virus (Fig. 6H). Six of nine bispecific antibodies tested neutralized the Beta variant similarly (within threefold variation) to wild-type SARS-CoV-2, despite the complete loss of binding of one component (CV521) in two of these antibodies and the reduced potency of at least one component (CV503/CV664/CV993) in all the others (Fig. 6H). For instance, a bispecific antibody combining CV503 and CV664 (CV503_664_EL) actually improved slightly in potency against the Beta variant, although CV503 and CV664 were both less effective against the variant as individual mAbs (Fig. 6I and fig. S7A). Two bispecific antibodies neutralized the Alpha, Beta, and Gamma variants, as well as the recently ascendant Delta variant, with little loss of potency compared to wild-type virus (Fig. 6H).

### Bispecific antibodies prevent disease mediated by wild-type or E484K SARS-CoV-2 in an in vivo model

**Fig. 7.**
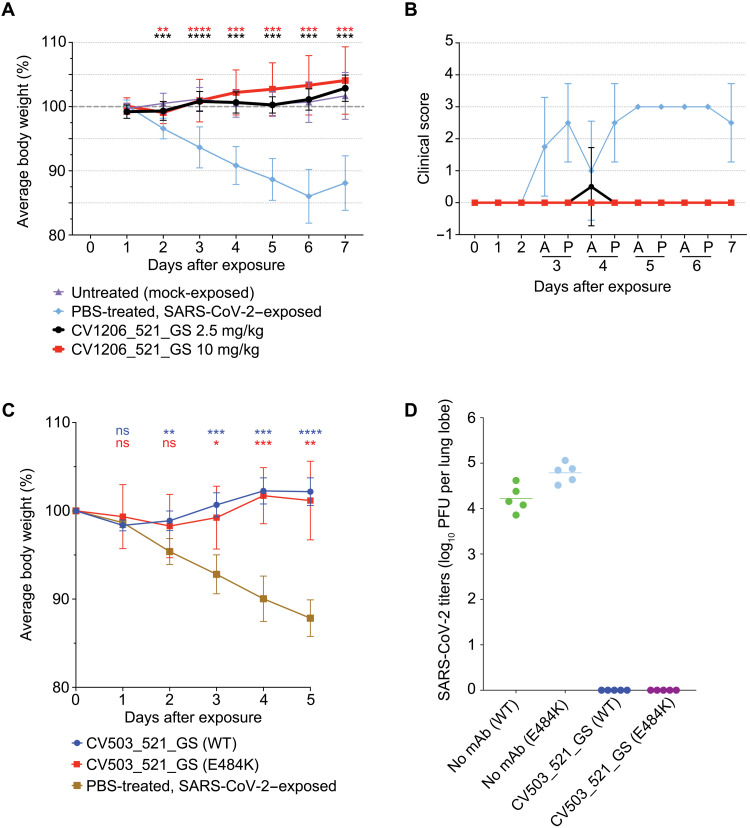
Bispecific antibodies prevent disease mediated by wild-type or E484K SARS-CoV-2 in a hamster model of infection. (**A**) Weight change is shown for hamsters that were administered with CV1206_521_GS intraperitoneally at a dose of 2.5 or 10 mg/kg, 24 hours before intranasal virus exposure at 5 log_10_ PFU (USA-WA1-A12/2020 strain). Differences between groups that were given the antibody versus PBS were determined using a mixed-effects repeated measures analysis with Dunnett’s multiple comparisons; **P < 0.01, ***P < 0.001, and ****P < 0.0001. *n* = 12 hamsters per group for days 0 to 3; *n* = 6 per group for days 4 to 7. Points represent means ± SD. (**B**) Blinded clinical scores assigned to hamsters throughout the course of disease are shown. A, a.m.; P, p.m. Points represent means ± SD. (**C**) Weight change is shown for hamsters that were administered with bispecific antibodies at 1 mg per hamster, 12 hours before intranasal virus exposure at 5 log_10_ PFU. The hamsters were infected with SARS-CoV-2 USA-WA1/2020 [WT (wild-type)] or E484K SARS-CoV-2 (E484K). Differences between groups that were given the antibody versus PBS were determined using a mixed-effects repeated measures analysis with Dunnett’s multiple comparisons; *P < 0.05, **P < 0.01, ***P < 0.001, and ****P < 0.0001. *n* = 5 hamsters per group. Points represent means ± SD. (**D**) Lung viral load was measured in antibody-treated hamsters exposed to SARS-CoV-2 USA-WA1/2020 (WT) or E484K SARS-CoV-2 (E484K) 5 days after infection. Bars show the mean.

We tested the bispecific antibodies for efficacy against SARS-CoV-2 infection in the well-established Syrian hamster model, because this model resembles features of severe COVID-19 in humans (Fig. 7) (*3*, *7*, *38*–*41*). In the first experiment, the bispecific antibodies were delivered intraperitonially at 2.5 or 10 mg/kg, followed by intranasal administration of 10^5^ plaque-forming units (PFU) of SARS-CoV-2 24 hours later. Change in body weight and a blinded clinical score were used to assess SARS-CoV-2–mediated disease. Consistent with previous reports, hamsters injected with phosphate-buffered saline (PBS) as sham treatment had a greater than 10% reduction in body weight through day 6 after infection, followed by a rebound in weight, whereas mock-exposed hamsters had no weight change (Fig. 7A) (*38*–*40*). SARS-CoV-2–exposed hamsters that had received CV1206_521_GS (2.5 or 10 mg/kg) had no weight loss through the week-long observation period, similar to the uninfected controls (*P* < 0.01 from days 2 to 7 relative to the sham-treated SARS-CoV-2 group) (Fig. 7A). No clinical signs (fig. S7B) were observed in hamsters in the mock-exposed group or in the virus-exposed groups that received either dose of CV1205_521_GS, with the exception of one hamster in the 2.5 mg/kg group that had a rapid respiratory rate on day 4, but then recovered (Fig. 7B). In contrast, rapid shallow breathing was observed in 7 of 12 hamsters in the sham-treated SARS-CoV-2–exposed group starting on day 3, and all remaining hamsters in this group developed clinical signs by day 5 through the end of the study (Fig. 7B). We confirmed the efficacy of CV1206_521_GS in preventing clinical disease caused by wild-type SARS-CoV-2 in the hamster model at an independent laboratory (fig. S7C). Next, we tested the in vivo efficacy of a potent bispecific antibody that neutralized the Beta variant, CV503_521_GS, against SARS-CoV-2 carrying a critical E484K variant mutation, which reduces the neutralization potency of many mAbs and convalescent plasma (*16*, *17*). CV503_521_GS was equally effective in vivo against wild-type SARS-CoV-2 and the virus carrying this mutation (Fig. 7C), matching our in vitro findings (Fig. 6H). Moreover, lung viral loads 5 days after infection were undetectable in hamsters treated with this bispecific antibody (Fig. 7D). We also tested an equimolar cocktail of the mAbs CV503 and CV521, as well as mAbs CV1206 and CV521, in the same model but could not distinguish between these cocktails and their corresponding bispecific antibodies because both were equally protective at the dose tested (fig. S7C). Nevertheless, the in vitro neutralization results with CV1206_521_GS (Fig. 6E) suggest that certain bispecific antibodies have higher potency than cocktails of the parent mAbs. Together, these results suggest that the bispecific antibodies are effective in preventing SARS-CoV-2–mediated disease in vivo, including disease caused by SARS-CoV-2 carrying a key variant mutation.

## DISCUSSION

The emergence of new SARS-CoV-2 variants (*10*–*13*) that appear to compromise vaccine efficacy and are resistant to many existing mAbs, including some currently in the clinic (*14*–*18*), necessitates further development of potent mAbs against the virus. In this study, we found that both plasmablasts and MBCs are capable of producing high-affinity, potent neutralizing antibodies that target diverse regions of the SARS-CoV-2 spike protein. These findings suggest that plasmablasts are a previously untapped source of potent mAbs targeting SARS-CoV-2 that should be interrogated further. A recent study showed that antibodies evolve over time after SARS-CoV-2 infection to acquire increased potency and breadth (*42*). The identification of plasmablasts that produce potent neutralizing antibodies, some of which are effective against SARS-CoV-2 variants of concern, suggests that the immune system is also capable of producing potent antibodies that control viral infection at an early stage. Although the precise interval between symptom onset and sample collection is unknown because of the nature of the study population, the B cells analyzed in this study were likely obtained during the later phase of an acute primary immune response, as evidenced by lower mutation frequencies within SARS-CoV-2–specific plasmablasts, the low frequency of mAbs that cross-reacted with spikes from previously circulating betacoronaviruses, and the presence of SARS-CoV-2–specific MBCs in circulation.

We did not observe unequivocal synergy among the antibody combinations tested, consistent with the paucity of studies demonstrating true synergy among SARS-CoV-2–specific mAbs (*9*). Nevertheless, antibody cocktails that target different regions of the spike protein offer the potential advantage of being more resistant to emerging variants. Antibody cocktails are the main format currently used in the clinic for treatment of SARS-CoV-2 (*43*, *44*). We explored the use of DVD-Ig bispecific antibodies as a potential alternative to antibody cocktails. Bispecific antibodies have recently been approved by the U.S. Food and Drug Administration (FDA) for treatment of hemophilia A (*45*) and acute lymphoblastic leukemia (*46*) and are under investigation for other malignancies (*47*) and for HIV (*48*–*50*). Bispecific antibodies offer three potential advantages over mAbs or mAb cocktails. First, bispecific antibodies can neutralize SARS-CoV-2 via mechanisms not available to mAbs, as demonstrated by the ability of CV1206_521_GS to cross-link adjacent spike proteins using its dual RBD and NTD specificities, which was associated with superior neutralization compared to a cocktail of the parent mAbs CV1206 and CV521. It will be of interest to confirm that bispecific antibodies can cross-link spike proteins on live SARS-CoV-2 and, as a result, potentially cross-link virions. Second, bispecific antibodies may be more resistant to emerging SARS-CoV-2 variants than single mAbs because they target multiple sites of the spike protein. For example, a recent study tested a bispecific antibody using a different format against SARS-CoV-2 variants of concern (*51*). This antibody showed similar or slightly improved in vitro potency against the Alpha and Gamma variants relative to wild-type SARS-CoV-2 but greater than 10-fold less potency against the Beta variant. Here, two bispecific antibodies tested retained the ability to neutralize the Alpha, Beta, Gamma, and Delta variants at near wild-type potency. Crucially, a potent bispecific antibody was efficacious against SARS-CoV-2 carrying a key variant mutation E484K in the hamster model. Both the bispecific antibodies and the mAb cocktails protected in vivo at the doses tested, so future dose de-escalation experiments should be performed to distinguish their in vivo potency. Third, from a clinical development standpoint, as single molecules, bispecific antibodies potentially offer practical and cost advantages over mAb cocktails (*52*). The DVD-Ig bispecific antibodies identified in this study that are of interest for clinical development will need to be evaluated for features of developability such as solubility, immunogenicity, and stability. Prior success in bringing DVD-Ig antibodies to at least four phase 2 clinical trials (*53*) suggests that the DVD-Ig format generally has a favorable developability profile.

This study has several limitations. First, we found that C135 (which binds away from the RBS) and several NTD-specific mAbs were neutralizing in authentic SARS-CoV-2 assays but not in the pseudovirus assay. These inconsistent results may be due to the overexpression of ACE2 in the cell line used in the pseudovirus assay, consistent with a recent study suggesting that ACE2 overexpression leads to an apparent reduction in neutralization of antibodies that do not directly prevent attachment of the SARS-CoV-2 spike to ACE2 (*54*). Going forward, it would be of interest to standardize pseudovirus neutralization assays with optimal cell lines and also to include at least one authentic SARS-CoV-2 neutralization assay when comparing the potency of antibodies from different sources. Second, we show that bispecific antibodies can prevent clinical signs in the hamster model, but further studies are required to determine the bioavailability of bispecific antibodies in the upper and lower airways and the degree to which they suppress viral replication in these tissues, particularly with respect to conventional mAbs that target SARS-CoV-2. Third, we cannot rule out that some of the antibodies with broader neutralization profiles were triggered by infection with SARS-CoV-2 carrying variant mutations. We examined the GISAID sequence database through the COVID-19 Viral Genome Analysis Pipeline (cov.lanl.gov) and did not find sequences from the Alpha, Beta, Gamma, and Delta variants in New York during the period of sample collection. However, there were multiple, independent introductions of virus variants into New York from Europe and other parts of the United States during that time period (*55*), leaving open the possibility that undetected variants may have been circulating.

In summary, we have isolated a panel of mAbs from plasmablasts and MBCs from COVID-19 convalescent individuals that target distinct regions of the SARS-CoV-2 spike protein. From this antibody panel, we designed bispecific antibodies that potently neutralize a range of SARS-CoV-2 variants of concern, including the currently dominant Delta variant. In the face of rapidly emerging SARS-CoV-2 variants that challenge our efforts to end the pandemic, our findings support the further exploration of bispecific antibodies that strategically combine antibody pairs as new tools to treat COVID-19.

## MATERIALS AND METHODS

### Study design

The study was designed to discover and characterize potent human monoclonal and bispecific antibodies against SARS-CoV-2 as potential tools for the prevention of COVID-19 and to guide vaccine design. The key procedures used to achieve this aim were high-throughput B cell screening, in vitro neutralization assays, the hamster in vivo model, and SPR for characterization of antibody affinity and specificity. Plasma and peripheral blood mononuclear cell (PBMC) samples were obtained from the New York Blood Center (NYBC) from 126 anonymous blood donors. Inclusion criteria included age ≥18 years, a diagnosis of symptomatic SARS-CoV-2 infection confirmed by RT-PCR, and a lack of COVID-19 symptoms for at least 2 weeks at the time of blood collection, according to FDA guidance. All donors signed the standard NYBC blood donor consent form that indicates that blood may be used for research. All 126 donors were included in the initial screen of plasma antibody reactivity to SARS-CoV-2, and 12 donors were selected for subsequent mAb screening based on plasma antibody reactivity to SARS-CoV-2. No randomization or blinding was performed in the analysis of human mAbs, plasma, or PBMC samples. Experimental replicates were performed as described in each figure legend.

### Sample processing

Whole blood remaining after infectious disease testing (3 to 7 ml per donor) was deidentified and provided for research purposes. All blood samples were collected during the month of April 2020. PBMCs were isolated by Ficoll-Paque density gradient centrifugation. After centrifugation, 1 to 2 ml of plasma from the top layer were removed, transferred to cryovial tubes, and stored at −80°C. PBMCs were recovered and washed with Dulbecco’s PBS and resuspended in 1 ml of CryoStor CS-10. PBMCs were kept at −80°C for 24 hours and then stored at −196°C in vapor-phase liquid nitrogen. PBMC and plasma samples were transported by courier to the National Institutes of Health (NIH) on dry ice (−78.5°C) where they were stored in vapor-phase liquid nitrogen and at −80°C, respectively.

### SARS-CoV-2 and SARS-CoV-1 protein antigens

The SARS-CoV-2 NTD (residues 14 to 305), RBD (residues 319 to 541), and ectodomain of the spike protein (residues 14 to 1213 with R682G/R683G/R685G/K986P/V987P mutations) (GenBank: QHD43416.1) were cloned into a customized pFastBac vector. The SARS-CoV RBD (residues 306 to 527) and ectodomain of the spike protein (residues 14 to 1195, with K968P/V969P mutations) (GenBank: ABF65836.1) were similarly cloned. The RBD and NTD constructs were fused with an N-terminal gp67 signal peptide and a C-terminal His_6_ tag. The spike ectodomain constructs were fused with an N-terminal gp67 signal peptide and a C-terminal BirA biotinylation site, thrombin cleavage site, foldon domain, and His_6_ tag. Recombinant bacmid DNA was generated using the Bac-to-Bac system (Life Technologies). Baculovirus was generated by transfecting purified bacmid DNA into Sf9 cells using FuGENE HD (Promega) and subsequently used to infect suspension cultures of High Five cells (Life Technologies) at a multiplicity of infection (MOI) of 5 to 10. Infected High Five cells were incubated at 28°C with shaking at 110 rpm for 72 hours for protein expression. The supernatant was then concentrated using a Centramate cassette (10-kDa molecular weight cutoff for RBD and NTD, and 30-kDa molecular weight cutoff for spike proteins; Pall Corporation). The proteins were purified by Ni–nitrilotriacetic acid, followed by size exclusion chromatography.

### Plasma binding to SARS-CoV-2 and other coronavirus antigens

Streptavidin-coated beads with different intensities of phycoerythrin (PE)–channel fluorescence (Spherotech, SVFA-2558-6K and SVFB-2558-6K) were incubated with the following biotinylated antigens: 10 μg/ml of Middle East respiratory syndrome (MERS) spike (Sino Biological, 40069-V08B), NL63 spike (Sino Biological, 40604-V08B), 229E spike (Sino Biological, 40605-V08B), HKU1 spike (Sino Biological, 40606-V08B), OC43 spike (Sino Biological, 40607-V08B), SARS-CoV-2 spike, SARS-CoV-2 RBD, SARS-CoV-2 NTD, SARS-CoV-1 spike, and SARS-CoV-1 RBD or CD4 (10 μg/ml) (*56*) as a control. SARS-CoV-2 and SARS-CoV-1 antigens were produced in-house as described above. Excess streptavidin sites were blocked with CD4 (10 μg/ml), and the beads were washed and mixed. The beads were stained with 1:50, 1:250, or 1:1250 plasma for 30 min at room temperature, washed, and stained with 2.5 μg/ml of goat anti-human IgG Alexa Fluor 647 (Jackson ImmunoResearch, 109-606-170), anti-human IgA Alexa Fluor 647 (Jackson ImmunoResearch, 109-606-011), or anti-human IgM Alexa Fluor 647 (Jackson ImmunoResearch, 109-606-129). The samples were read with the iQue Screener Plus (IntelliCyt) high-throughput flow cytometer, and FACS data were analyzed with FlowJo. Data from titrations were analyzed by calculating the area under the curve (AUC) for the titration and subtracting the AUC of the negative control antigen.

### Fluorescence reduction neutralization assay

Assays for determining neutralizing titers with authentic SARS-CoV-2 [2019-nCoV/USA-WA1-A12/2020 from the U.S. Centers for Disease Control and Prevention (CDC)] were performed at the NIH National Institute of Allergy and Infectious Diseases Integrated Research Facility using a fluorescence reduction neutralization assay (FRNA), as previously described (*57*). This assay was performed by incubating a fixed volume of virus (0.5 MOI) with the antibodies for 1 hour at 37°C before adding to Vero E6 cells (Biodefense and Emerging Infections Research Resources Repository, NR-596) plated in 96-well plates. After addition to Vero E6 cells, the virus was allowed to infect the cells and propagate for 24 hours at 37°C/5% CO_2_, at which time the cells were fixed with neutral buffered formalin. After fixation, the cells were permeabilized with Triton X-100 and probed with a SARS-CoV/SARS-CoV-2 nucleoprotein-specific rabbit primary antibody (Sino Biological, 40143-R001) followed by an Alexa Fluor 647–conjugated secondary antibody (Life Technologies, A21245). Cells were counterstained with Hoechst nuclear stain (Life Technologies, H3570). Cells in four fields per well were counted with each field containing at least 1000 cells. Each dilution step of test sample(s) was run in quadruplicate. Plates were read and quantified using an Operetta high-content imaging system (PerkinElmer). Antibodies were screened using a twofold serial 12-step dilution. The lower limit of detection was either 1:20 or 1:40 depending on the dilution series. Assays were controlled using a spike protein–specific antibody as positive control in addition to virus-only and uninfected cell controls.

Percent neutralization was calculated as 100 − [(percent infected cells in well of interest/average percent infected cells from six virus-only wells in matched plate) × 100]. The average percent neutralization of quadruplicate samples was determined per antibody dilution point. Plasma and mAb IC_50_ values were estimated using an automated curve fitting script that fit four-parameter logistic model nonlinear regressions under 22 different combinations of model constraints and parameter starting values, or if those models failed to converge, then a quadratic regression model was used. All 22 combinations of the logistic nonlinear regression were attempted, and if any succeeded, then the best fitting model was selected using its *R*^2^ value. Quadratic linear regression was only used if all 22 logistic nonlinear regressions failed. Regardless of the model fit, IC_50_ was estimated using interpolation to find the concentration or dilution that generated 50% neutralization.

### Authentic SARS-CoV-2 neutralization assay (Scripps)

Vero E6 cells were seeded in 96-well half-well plates at about 8000 cells per well in a total volume of 50 μl of complete Dulbecco’s modified Eagle’s medium [DMEM; supplemented with 10% heat-inactivated serum, 1% GlutaMAX, and 1% penicillin/streptomycin (P/S)] the day before the in-cell enzyme-linked immunosorbent assay. Virus (500 PFU per well) and antibodies were mixed, incubated for 30 min, and added to the cells. The transduced cells were incubated at 37°C for 24 hours. Each treatment was tested in duplicate. The medium was removed and disposed of appropriately. Cells were fixed by immersing the plate into 4% formaldehyde for 1 hour before washing three times with PBS. The plate was then either stored at 4°C or gently shaken for 30 min with 100 μl per well of permeabilization buffer (consisting of PBS with 1% Triton X-100). All solutions were removed and then 100 μl of 3% bovine serum albumin (BSA) was added, followed by room temperature incubation for 2 hours.

Primary antibodies against the spike protein were generated from a high-throughput screen of samples from a convalescent, COVID-19 cohort (CC) (*7*). A mix of primary antibodies consisting of CC6.29, CC6.33, L25-dP06E11, CC12.23, and CC12.25, in an equimolar ratio, was used next. The primary antibody mixture was diluted in PBS with 1% BSA to a final concentration of 2 μg/ml. The blocking solution was removed and washed thoroughly with wash buffer (PBS with 0.1% Tween 20). The primary antibody mixture, 50 μl per well, was incubated with the cells for 2 hours at room temperature. The plates were washed three times with wash buffer.

Secondary antibody [Jackson ImmunoResearch, Peroxidase AffiniPure Goat Anti-Human IgG (H+L), 109-035-088] diluted to 0.5 mg/ml in PBS with 1% BSA was added at 50 μl per well and incubated for 2 hours at room temperature. The plates were washed six times with wash buffer. Horseradish peroxidase substrate (Roche, 11582950001) was freshly prepared as follows: Solution A was added to solution B in a 100:1 ratio and stirred for 15 min at room temperature. The substrate was added at 50 μl per well, and chemiluminescence was measured in a microplate luminescence reader (BioTek, Synergy 2).

The following method was used to calculate the percentage neutralization of SARS-CoV-2. First, we plotted a standard curve of serially diluted virus (3000, 1000, 333, 111, 37, 12, 4, and 1 PFU) versus relative light units (RLU) using four-parameter logistic regression (GraphPad Prism version 8) below

$$y=a+\frac{b-a}{1+{\left(\frac{x}{x_{0}}\right)}^{c}}$$

where *a*, *b*, *c*, and *x*_0_ are parameters fitted by standard curve. RLU value and PFU value were used for *y* and *x*, respectively.

To convert sample RLU into PFU, use the equation below (if *y* < *a*, then *x* = 0)

$$x=x_{0}{\text{log}}_{c}\frac{b-y}{y-a}$$

Percentage neutralization was calculated by the following equation

$$\%\text{Neut}=100\times \frac{\text{VC}-\text{nAb}}{\text{VC}-\text{CC}}$$

where VC is the average of vehicle-treated control, CC is the average of cell-only control, and nAb is the neutralizing antibody. PFU value was used for each variable indicated.

To compute neutralization IC_50_, logistic regression (sigmoidal) curves were fit using GraphPad Prism. Means and SDs are displayed in the curve fit graphs and were also calculated using GraphPad Prism.

### Murine leukemia virus pseudovirus neutralization assay

Pseudovirus preparation and assay were performed as previously described with minor modifications (*7*). Pseudovirions were generated by cotransfection of human embryonic kidney (HEK) 293T cells with plasmids encoding murine leukemia virus (MLV)–gag/pol, MLV-CMV-Luciferase, and SARS-CoV-2 spike (GenBank: MN908947) with an 18–amino acid truncation at the C terminus. Supernatants containing pseudotyped virus were collected 48 hours after transfection and frozen at −80°C for long-term storage. Pseudovirus-neutralizing assay was carried out as follows. Twenty-five microliters of mAbs serially diluted in DMEM with 10% heat-inactivated fetal bovine serum (FBS), 1% Q-Max, and 1% P/S were incubated with 25 μl of SARS-CoV-2 pseudovirus at 37°C for 1 hour in 96-well half-well plates (Corning, 3688). After the incubation, 10,000 HeLa-hACE2 cells were generated by lentivirus transduction of wild-type HeLa cells and enriched by FACS using biotinylated SARS-CoV-2 RBD conjugated with streptavidin–Alexa Fluor 647 (Thermo Fisher Scientific, S32357) that were added to the mixture with dextran (20 μg/ml) (Sigma-Aldrich, 93556-1G) for enhanced infectivity. At 48 hours after incubation, the supernatant was aspirated, and HeLa-hACE2 cells were then lysed in luciferase lysis buffer [25 mM Gly-Gly (pH 7.8), 15 mM MgSO_4_, 4 mM EGTA, and 1% Triton X-100]. Bright-Glo (Promega, PR-E2620) was added to the mixture following the manufacturer’s instruction, and luciferase expression was read using a luminometer. Patient samples were tested in duplicate, and assays were repeated at least twice for confirmation. Neutralization inhibitory dose (ID_50_) titers or IC_50_ values were calculated using “One-Site Fit LogIC50” regression in GraphPad Prism 9.

$$100\times (1-\frac{\text{RLUs of sample}-\text{Average RLUs of background}}{\text{Average RLUs of virus control}-\text{Average RLUs of background}})$$

### SARS-CoV-2 variant plasmid generation

The Alpha (B.1.1.7), Beta (B.1.351), and Gamma (P.1) plasmids were generated from the pCDNA3.3-SARS2-spike-WT(D18) plasmid with the mutations and primers shown in table S3. Each fragment containing two mutations on the flanking side was PCR-amplified and purified by gel electrophoresis. Fragments were then PCR-amplified into one long piece and ligated into the pCDNA3.3 backbone digested with Bam HI (New England Biolabs) and Xho I (New England Biolabs).

### Lentivirus neutralization assay

Spike-containing lentiviral pseudovirions were produced by cotransfection of packaging plasmid pCMVdR8.2, transducing plasmid pHR′ CMV-Luc, spike plasmid from SARS-CoV-2 (Wuhan-1, D614G, B.1.1.7, B.1.351) with transmembrane serine protease 2 (TMPRSS2) into 293T cells using FuGENE 6 transfection reagent (Promega) (*58*–*60*). Alpha/B.1.1.7 virus contained the following spike mutations: del-H69–V70, del-Y144, N501Y, A570D, D614G, P681H, T716I, S982A, and D1118H. Beta/B.1.351 virus contained the following spike mutations: L18F, D80A, D215G, del-L242_244, R246I, K417N, E484K, N501Y, D614G, and A701V. Gamma/P.1 virus contained the following spike mutations: L18F, T20N, P26S, D138Y, R190S, K417T, E484K, N501Y, D614G, H655Y, T1027I, and V1176F. Delta/B.1.617.2 virus contained the following spike mutations: T19R, G142D, del156-157, R158G, L452R, T478K, D614G, P681R, and D950N. 293T-ACE2 cells, provided by M. Farzan, were plated into 96-well white/black IsoPlates (PerkinElmer) at 5000 cells per well the day before transdution of SARS-CoV-2. Serial dilutions of mAbs were mixed with titrated pseudovirus, incubated for 45 min at 37°C, and added to 293T-ACE2 cells in triplicate. After 2 hours of incubation, wells were replenished with 100 ml of fresh media. Cells were lysed 72 hours later, and luciferase activity was measured with MicroBeta (PerkinElmer). Percent neutralization and neutralization IC_50_ values were calculated using GraphPad Prism 8.0.2.

### Antibody binding to cell surface expressed full-length SARS-CoV-2 spike proteins

HEK293T cells were transiently transfected with plasmids encoding full-length SARS-CoV-2 spike variants using Lipofectamine 3000 (L3000-001, Thermo Fisher Scientific) following the manufacturer’s protocol. After 40 hours, the cells were harvested and incubated with bispecific antibodies and mAbs (1 μg/ml) for 30 min. After incubation with the antibodies, the cells were washed and incubated with an allophycocyanin (APC)–conjugated anti-human IgG (709-136-149, Jackson ImmunoResearch Laboratories) for another 30 min. The cells were then washed and fixed with 1% paraformaldehyde (PFA; 15712-S, Electron Microscopy Sciences). The samples were then acquired in a BD LSRFortessa X-50 flow cytometer (BD Biosciences) and analyzed using FlowJo (BD Biosciences).

### Optofluidics-based identification of SARS-CoV-2–specific mAbs

Cryopreserved PBMCs were thawed and stained with the following panel: LIVE/DEAD Fixable Aqua (Thermo Fisher Scientific, L34966) or 4′,6-diamidino-2-phenylindole (BD Biosciences, BD564907), CD14–Brilliant Violet (BV) 510 (BioLegend, 301842), CD3-BV510 (BioLegend, 317332), CD56-BV510 (BioLegend, 318340), CD19–Energy-Coupled Dye (Beckman Coulter, IM2708U), IgA–Alexa Fluor 647 (Jackson ImmunoResearch, 109-606-011), IgD-PE-Cy7 (BD Biosciences, BD 561314), and IgM–Peridinin-Chlorophyll-Protein–Cy5.5 (BD Biosciences, BD561285), CD27–Alexa Fluor 488 (BioLegend, 393204), and CD38-APC-Cy7 (BioLegend, 303534). The cells were sorted using the BD FACSAria IIIu in a biosafety level 3 facility and gated on live CD19^+^CD14^−^CD3^−^CD56^−^CD27^++^CD38^++^ (plasmablasts) or live CD19^+^CD14^−^CD3^−^CD56^−^CD27^+^IgM^−^IgD^−^IgA^+^/IgA^−^ (MBCs). Plasmablasts were sorted into the Plasma Cell Survival Medium (Berkeley Lights) and screened in the Beacon device (Berkeley Lights) using standard workflows in the Cell Analysis Suite program. Briefly, sorted plasmablasts were loaded onto an OptoSelect 11k chip and transported as single cells using opto-electropositioning (OEP) light cage technology into nanoliter-volume pens. The cells were screened in a 30-min time course assay for secretion of antibodies that bound to 7-μm streptavidin beads (Spherotech) coated with 10 μg/ml of SARS-CoV-2 spike or RBD. Antibody binding to the beads was detected with 2.5 μg/ml of goat anti-human IgG–Alexa Fluor 647 (Jackson ImmunoResearch, 109-606-170), goat anti-human IgA-Cy3 (Jackson ImmunoResearch, 109-166-011), and goat anti-human IgM–Alexa Fluor 488 (Jackson ImmunoResearch, 109-546-129), which were added along with the beads during the assay. B cells of interest were selected for RNA extraction and production of complementary DNA (cDNA) in two ways. First, under the Cell Unload protocol, B cells of interest were exported individually using OEP light cages into 96-well plates for single-cell RT-PCR and total cDNA amplification with the Opto Plasma B Discovery cDNA Synthesis Kit (Berkeley Lights). Alternatively, the BCR Unload protocol was used, where RNA capture beads were imported into the nanopens containing B cells of interest, followed by lysis of the cells and reverse transcription in situ to generate cDNA on the beads. The RNA capture beads were exported from the Beacon for total cDNA amplification off-chip. After cDNA amplification from either approach, gene-specific PCR was performed as previously described (*61*). MBCs were plated into 96-well plates at 2500 cells per well and cultured in a proprietary cytokine cocktail (Berkeley Lights) for 6 days, followed by screening of supernatants for binding to beads coated with 10 μg/ml of SARS-CoV-2 spike or RBD using the iQue Screener. B cells from wells of interest were loaded onto the Beacon for single-cell screening, using the same workflows described above.

### mAb sequence analysis and production

Antibody heavy and light chains were PCR-amplified and sequenced as previously described (*61*, *62*) Sequence analysis, including the determination of the *VH* and *VL* genes and percentage of somatic mutations, was performed using the International Immunogenetics Information System database (*63*). Antibody V_H_ or V_L_ sequences were cloned into plasmids containing an IgG1 or relevant light chain backbone (GenScript) and used to transfect Expi293 cells (Thermo Fisher Scientific). Recombinant IgG was purified using HiTrap Protein A columns (GE Healthcare Life Sciences). For IgA1 or IgA2 antibodies, V_H_ sequences were also cloned into plasmids containing the IgA1 or IgA2 constant region (GenScript). Recombinant IgA was expressed without a J chain (to express only monomeric IgA) and purified using columns containing the CaptureSelect IgA Affinity Matrix (Thermo Fisher Scientific). To produce antibody Fabs, heavy chain plasmids encoding only the V_H_ and C_H_1 (domain 1 of constant region of the immunoglobulin heavy chain) were synthesized and used to transfect Expi293 cells along with light chain plasmids. Fab purification (including for the CV503 crystal structure) was performed with the CaptureSelect KappaXP Affinity Matrix or CaptureSelect LC-lambda (Hu) Affinity Matrix (Thermo Fisher Scientific).

### mAb binding to SARS-CoV-2 and other coronavirus antigens

For screening of binding to different coronavirus antigens, streptavidin beads were coated as described above with the following biotinylated antigens: 10 μg/ml of MERS spike (Sino Biological, 40069-V08B), NL63 spike (Sino Biological, 40604-V08B), 229E spike (Sino Biological, 40605-V08B), HKU1 spike (Sino Biological, 40606-V08B), OC43 spike (Sino Biological, 40607-V08B), SARS-CoV-2 NTD, SARS-CoV-1 spike, and SARS-CoV-1 RBD (see above for details of production) or CD4 (10 μg/ml) as a control. The beads were stained with 10 μg/ml of each antibody for 30 min at room temperature, washed, and stained with goat anti-human IgG Alexa Fluor 647 (2.5 μg/ml) (Jackson ImmunoResearch, 109-606-170). For screening of binding to SARS-CoV-2 spike and RBD, streptavidin beads were coated with 10 μg/ml of SARS-CoV-2 spike, RBD, or CD4 (*56*) as a negative control. The beads were incubated with 12 dilutions of the mAbs for 30 min at room temperature, washed, and stained with goat anti-human IgG Alexa Fluor 647 (2.5 μg/ml). All samples were read with the iQue Screener Plus (IntelliCyt) high-throughput flow cytometer, and FACS data were analyzed with FlowJo. Data for binding to coronavirus antigens were analyzed by subtracting median fluorescence intensity (MFI) values of the negative control antigen from the MFI values of each antibody. Data for binding to SARS-CoV-2 spike and RBD were analyzed by calculating the AUC for each antibody without subtraction of negative control antigen.

### Antibody kinetics

All kinetics experiments were performed with the Carterra LSA. An HC30M chip (Carterra) was primed with filtered and degassed Hepes-buffered saline Tween-EDTA (HBSTE). The chip was activated with a mixture of 400 mM 1-ethyl-3-(3-dimethylaminopropyl)carbodiimide hydrochloride and 100 mM *N*-hydroxysuccinimide (Thermo Fisher Scientific), followed by coupling with goat anti-human IgG Fc (50 μg/ml) (Jackson ImmunoResearch, 109-005-098) in 10 mM sodium acetate (pH 5.0), and blocking with 1 M ethanolamine (pH 8.5). Next, 1 or 5 μg/ml concentration of each antibody in HBSTE was printed onto a discrete spot on the chip. A series of seven increasing concentrations of monomeric SARS-CoV-2 RBD (up to 500 nM) or NTD (up to 1.5 μM) was added to the antibody spots sequentially; association was performed for 5 min and dissociation for 15 min to obtain kinetics data. The results were analyzed as nonregenerative kinetics data using the Kinetics Software (Carterra) to obtain association rate (*K*_a_), dissociation rate (*K*_d_), and *K*_D_ values.

### Epitope binning

Epitope binning experiments were performed with the Carterra LSA. An HC200M chip (Carterra) was primed with filtered and degassed HBSTE and 0.05% BSA. The chip was activated as described above, followed by direct coupling with 10 μg/ml of mAbs of interest in acetate buffer (pH 4.5) to discrete spots on the chip and blocking with 1 M ethanolamine (pH 8.5). Monomeric 50 nM SARS-CoV-2 RBD or 500 nM SARS-CoV-2 NTD was added to the antibody spots, followed by addition of 10 μg/ml of the sandwiching mAb or protein. Regeneration after each sandwiching antibody was performed with 10 mM glycine (pH 2.0). Binning data were analyzed using the Epitope Software (Carterra).

### Design of DVD-Ig bispecific antibodies

The mAbs CV503, CV521, CV664, CV993, CV1182, and CV1206 were used to create bispecific DVD-Ig antibodies. The antibodies were created as previously described (*37*) by synthesizing plasmids that had the heavy chains or the light chains of two antibodies in tandem. For antibodies ending in _GS (Ab1_Ab2_GS), both heavy chains and both light chains were connected by a GGGGSGGGGSGGGG linker, with the first antibody making up the outer variable domains and the second antibody making up the internal domains, closer to the Fc. For antibodies ending in _EL (Ab1_Ab2_EL), the linkers were based on elbow regions between the variable region and constant region: For heavy chains, the sequence ASTKGPSVFPLAP was used. For light chains, the linker sequence QPKAAPSVTLFPP was used when the outer variable domain was of the lambda isotype, and the sequence TVAAPSVFIFPP was used when the outer variable domain was a kappa isotype.

### Size exclusion chromatography

To test bispecific antibody purity, each of the antibodies was run on a Superdex 200 column (Increase 10/300 GL, Cytiva) with the AKTA pure chromatography system. The column was preequilibrated with PBS. Thirty microliters of the antibody sample (at 0.5 mg/ml) was applied onto the column, and the column was eluted with PBS at 0.75 ml/min. Elution profiles were recorded with Unicorn software (Cytiva).

### Structure modeling

Epitope bins represented by C135 (PDB ID: 7K8Z), S309 (PDB ID: 6WPS), ACE2 (PDB ID: 6M0J), CR3022 (PDB ID: 6W41), as well as NTD-specific antibody 4-8 were modeled onto a SARS-CoV-2 spike protein (PDB ID: 7C2L). Residues with a buried surface area > 0 Å^2^, as calculated by the Proteins, Interfaces, Structures and Assemblies (PISA) program (*64*), were used for defining the epitopes. The epitope of antibody 4-8 refers to an approximate area according to Liu *et al*. (*5*) as coordinates of the complex structure are not publicly available.

### Crystallization and structural determination

Expression and purification of SARS-CoV-2 RBD used for crystallization is similar to the method described in the “SARS-CoV-2 and SARS-CoV-1 protein antigens” section, except that a truncated version of the SARS-CoV-2 RBD (residues 333 to 529) was used. The CV503 Fab, COVA1-16 Fab, and SARS-CoV-2 RBD were all stored in TBS buffer [20 mM tris (pH 7.4) and 150 mM NaCl]. A complex of CV503 with RBD and COVA1-16 was formed by mixing each of the protein components in an equimolar ratio and incubating overnight at 4°C without further purification steps. The protein complex was adjusted to 14 mg/ml in TBS buffer and screened for crystallization using the 384 conditions of the JCSG Core Suite (QIAGEN) on a robotic CrystalMation system (Rigaku) at the Scripps Research Institute. Crystallization trials were setup by the vapor diffusion method in sitting drops containing 0.1 μl of protein and 0.1 μl of reservoir solution. Optimized crystals were then grown in 0.1 M sodium citrate (pH 4.2), 1 M lithium chloride, and 9% (w/v) polyethylene glycol 6000. Crystals appeared on day 7, harvested on day 10 by soaking in reservoir solution supplemented with 15% (v/v) ethylene glycol, and then flash-cooled and stored in liquid nitrogen until data collection. Diffraction data were collected at cryogenic temperature (100 K) at the Stanford Synchrotron Radiation Lightsource on Scripps/Stanford beamline 12-1 with a beam wavelength of 0.97946 Å and processed with HKL2000 (*65*). Structures were solved by molecular replacement using Phaser (*66*) with PDB 7JMW for RBD and COVA1-16 (*34*), whereas the model of CV503 was generated by Repertoire Builder (https://sysimm.org/rep_builder**/**) (*67*). Iterative model building and refinement were carried out in Coot (*68*) and PHENIX (*69*), respectively.

### Single-particle nsEM

The bispecific antibodies were incubated with SARS-2 CoV 6P Mut7 at equal molar ratios for 30 min at room temperature. The complexes were diluted to 0.03 mg/ml in 1× TBS (pH 7.4) and applied to plasma-cleaned (argon/oxygen mix) copper mesh grids. Uranyl formate at 2% was applied to the grid for 55 s and then blotted off. Datasets were collected with the FEI Tecnai Spirit (120 keV, ×56,000 magnification) paired with a FEI Eagle (4 k by 4 k) charge-coupled device camera. Data collection details include a defocus value −1.5 μm, a pixel size of 2.06 Å per pixel, and a dose of 25 *e*^−^/Å^2^. Data collection automation was achieved with the Leginon (*70*) software, and resulting images were stored in the Appion (*71*) database. Complexed single particles were picked using DoGpicker (*72*) and stacked with a box size of 300 pixels. RELION 3.0 (*73*) was used for 2D and 3D classifications and final refinements. UCSF Chimera (*74*) enabled map segmentation and model docking.

### Hamster study (NIH)

The SARS-CoV virus used for these studies was the 2019-nCoV/USA-WA1-A12/2020 isolate of SARS-CoV-2 provided by the U.S. CDC that was expanded in Vero E6 cells. Virus sequence was consistent with the published sequence for this isolate. Forty-eight golden Syrian hamsters (*Mesocricetus auratus*; about 6 weeks old, individually housed) sourced from Envigo were randomly assigned to four groups of 12 animals each with equal numbers of males and females and a randomized treatment order. The study was blinded to the staff who handled the animals. Because of safety requirements of the biocontainment laboratory, hamsters were chemically restrained with 4 to 5% isoflurane for all manipulations. A single treatment of each dose (2.5 and 10 mg/kg) of the CV1206_521_GS antibody or PBS was administered to the respective group by intraperitoneal inoculation 24 hours before intranasal SARS-CoV-2 (or mock-virus) exposure. Groups of hamsters were then exposed intranasally to SARS-CoV-2 diluted in 100 μl (total volume split equally between nostrils) of DMEM containing 2% heat-inactivated FBS to the desired concentration at the 5 log_10_ PFU or mock inoculum (diluent). The inocula were back-titered in real time to determine the dose received. The actual dose was 5.13 log_10_ PFU as quantified by plaque assay. After exposure, all hamsters were weighed and monitored for up to 7 days. Animals were observed at least once daily by staff with experience in assessing distress in hamsters and assigned a clinical score.

### Hamster study (Scripps)

As previously described (*7*), 8-week-old Syrian hamsters were given an intraperitoneal antibody injection 12 hours before infection. Hamsters were infected through intranasal installation with 10^5^ total PFU per animal of SARS-CoV-2 (USA-WA1/2020) or recombinant SARS-CoV-2 E484K in 100 μl of DMEM. Hamsters were then weighed for the duration of the study. At day 5 after infection, animals were euthanized, and lungs were harvested for plaque live virus assays and histology. The research protocol was approved and performed in accordance with Scripps Research Institutional Animal Care and Use Committee (IACUC) protocol no. 20-0003.

### Viral load measurements

SARS-CoV-2 titers were measured by homogenizing organs in DMEM and 2% fetal calf serum using 100-μm cell strainers (Myriad, 2825-8367). Homogenized organs were titrated 1:10 over six steps and layered over HeLa-ACE2 cells and then incubated at 34°C for 24 hours. The cells were fixed with 4% PFA for 1 hour and then labeled with SARS-CoV-2 polyclonal plasma diluted in Perm/Wash buffer (BD Biosciences, 554723). The cells were then labeled with the Peroxidase AffiniPure Goat Anti-Human IgG, F(ab′)_2_ (Jackson ImmunoResearch, 109-035-097), stained using TrueBlue substrate (SeraCare, 5510-0030), and subsequently counted.

### Statistical analysis

Associations between plasma binding to SARS-CoV-2 spike, RBD, NTD, and plasma neutralization, as well as the correlation between plasma and mAb neutralization of the different donors, were evaluated using a two-tailed Spearman’s rank correlation. Comparisons of characteristics between plasmablasts and MBCs were performed with the Mann-Whitney *U* test. Synergy was assessed using Loewe’s additivity through the web application SynergyFinder v2.0 (https://synergyfinder.fimm.fi/) (*75*).

## Notes

View/request a protocol for this paper from *Bio-protocol*.
