# Supplementary material for: Bispecific antibodies targeting distinct regions of the spike protein potently neutralize SARS-CoV-2 variants of concern
Source: Sci Transl Med. 2021 Sep 14;13(616):eabj5413. doi: 10.1126/scitranslmed.abj5413 (PMC8651051; doi:10.1126/scitranslmed.abj5413)
Supplement: Supplementary file 1 — Materials and Methods Figs. S1 to S7 Tables S1 to S3 [file scitranslmed.abj5413_sm.pdf]

Science Translational Medicine
AAAS

# Supplementary Materials for

## Bispecific antibodies targeting distinct regions of the spike protein potently neutralize SARS-CoV-2 variants of concern

Hyeseon Cho *et al.*

Corresponding author: Peter D. Crompton, pcrompton@niaid.nih.gov; Joshua Tan, tanj4@nih.gov

*Sci. Transl. Med.* **13**, eabj5413 (2021)
DOI: 10.1126/scitranslmed.abj5413

**The PDF file includes:**

Materials and Methods
Figs. S1 to S7
Tables S1 to S3

**Other Supplementary Material for this manuscript includes the following:**

Data file S1

## MATERIALS AND METHODS

### Expression and purification of COVA1-16 Fab

The heavy chains (HC) and light chains (LC) of COVA1-16 were cloned into phCMV3. The plasmids were transiently co-transfected into ExpiCHO cells at a ratio of 2:1 (HC:LC) using ExpiFectamine CHO Reagent (Thermo Fisher Scientific) according to the manufacturer's instructions. The supernatant was collected at 10 days post-transfection. The Fabs were purified with a CaptureSelect CH1-XL Affinity Matrix (Thermo Fisher Scientific) followed by size exclusion chromatography on a HiLoad Superdex 200 pg column (GE Healthcare), and buffer exchanged into 20 mM Tris-HCl pH 7.4 and 150 mM NaCl.

### Antibody synergy experiments

Five antibodies, CV503, CV521, CV664, CV993 and CV1182, were tested for synergy in neutralizing authentic SARS-CoV-2 (FRNA assay). In the initial screens, each antibody was tested at twice the estimated neutralization half-maximal inhibitory concentration ($IC_{50}$) value (Dose 1) and at the estimated $IC_{50}$ value (Dose 2) in various combinations. Two combinations were explored further: CV503 and CV664, as well as CV664 and CV993. For each pair, each antibody was tested at 5 different concentrations in a checkerboard combination arrangement, along with single antibody controls. Three pairs: CV503 and CV664, CV664 and CV993, and CV1206 and CV521, were also further tested for synergy in the authentic SARS-CoV-2 (Scripps) or pseudovirus assays.

### SDS-PAGE

For each antibody, 2.5 to 5 µg was loaded onto a NuPAGE 4-12% gel (Thermo Fisher Scientific) and run at 100V for 2 to 3 hours. The gel was stained with PageBlue Staining Solution (Thermo Fisher Scientific) and destained with multiple rinses of water. Imaging was done using the LI-COR Odyssey Fc Imaging System, with emission set at 700 nm.

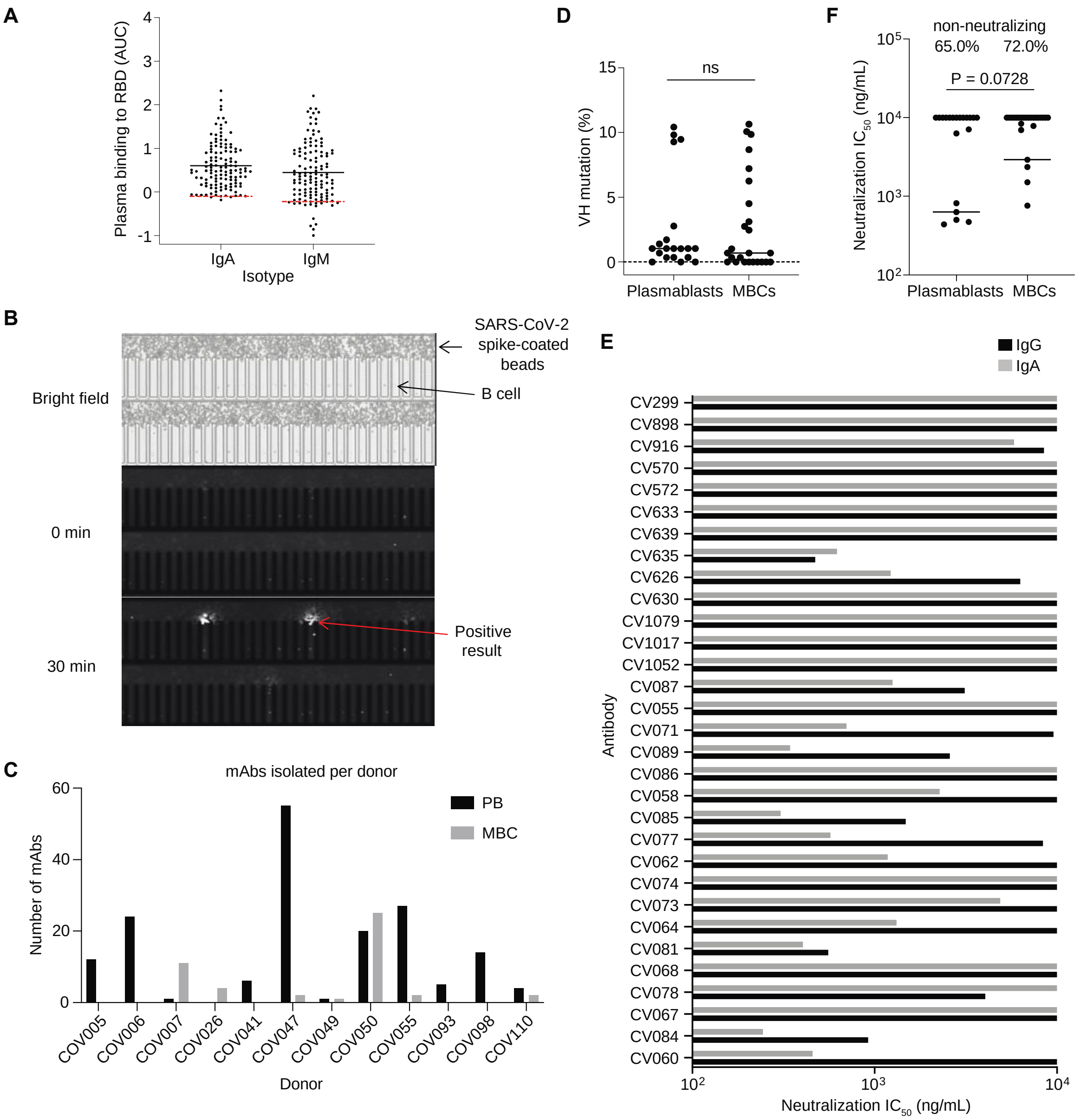


**Fig. S1. Characterization of mAbs isolated from SARS-CoV-2 convalescent donors.**

(A) Plasma IgA and IgM reactivity of coronavirus disease 2019 (COVID-19) convalescent donors to severe acute respiratory syndrome coronavirus 2 (SARS-CoV-2) receptor-binding domain (RBD) is shown (*N* = 1 experiment). The red dotted lines show the mean area under the curve (AUC) of 11 COVID-19-naïve donors in the United States as a baseline. AUC values are shown after subtraction of the negative control antigen. Black bars indicate mean AUC of the convalescent donors.

(B) An image of nanopens containing B cells is shown. The lower panel shows positive fluorescence signals from binding of secreted antibodies to SARS-CoV-2 spike protein-coated beads.

(C) Number of mAbs isolated per donor are shown, divided by cell type.

(D) VH mutations in mAbs isolated from donor COV050 are shown. Bars show median; data were analyzed using a Mann-Whitney U-test. ns, not significant.

(E) Authentic SARS-CoV-2 neutralization (FRNA) potency of IgG and IgA forms of the same mAbs (originally isolated as IgA) is shown (*N* = 1 experiment). Data are shown as neutralization $IC_{50}$ concentrations.

(F) Neutralization potency of antibodies isolated from donor COV050 is shown by cell type. Top values indicate percentages of non-neutralizing antibodies. Bars indicate median; data were analyzed using a Mann-Whitney U-test with non-neutralizing antibodies excluded from calculation (*N* = 1 experiment).

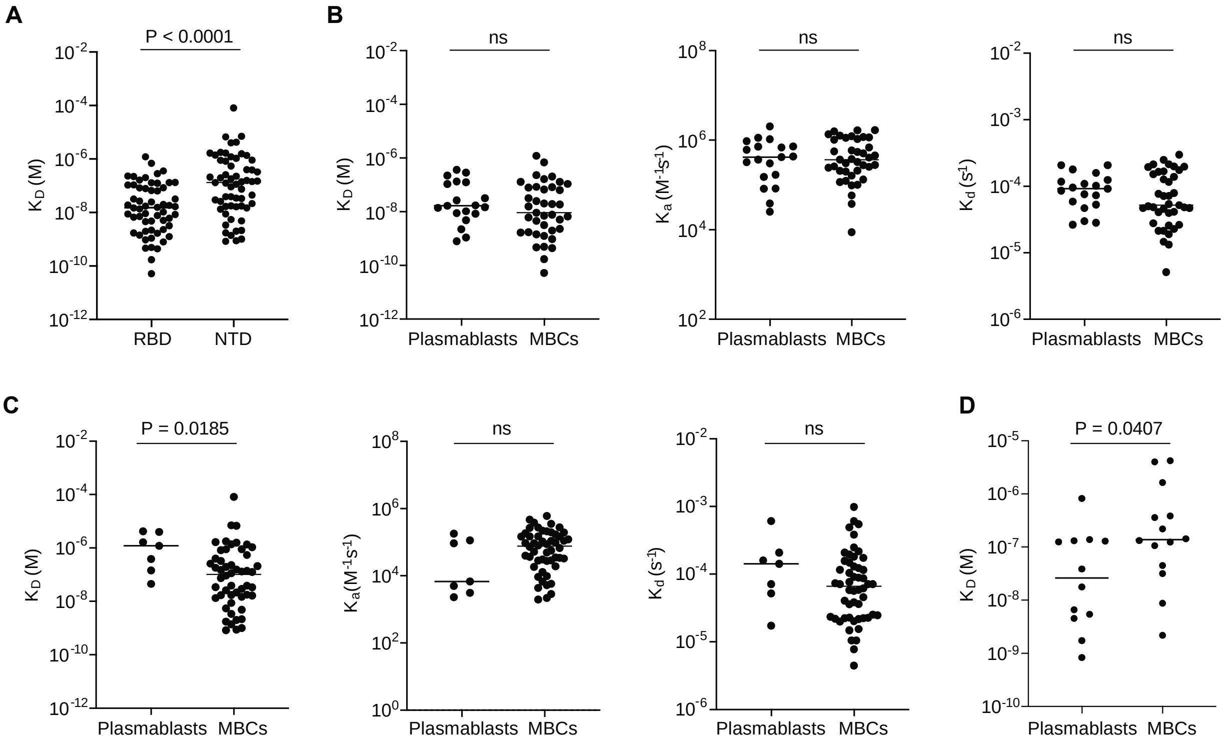


**Fig. S2. Comparison of affinity of antibodies from plasmablasts and memory B cells (MBCs).**

(A) A comparison of affinity of SARS-CoV-2 RBD-specific and N-terminal domain (NTD)-specific antibodies is shown (representative of $N = 2$ experiments). Bars show median; data were analyzed using a Mann-Whitney U-test.

(B) A comparison of affinity ($K_D$), association rates ($K_a$) and dissociation rates ($K_d$) of SARS-CoV-2 RBD-specific antibodies is shown (representative of $N = 2$ experiments). Bars show median; data were analyzed using a Mann-Whitney U-test. ns, not significant.

(C) A comparison of affinity ($K_D$), association rates ($K_a$) and dissociation rates ($K_d$) of SARS-CoV-2 NTD-specific antibodies is shown (representative of $N = 2$ experiments). Bars show median; data were analyzed using a Mann-Whitney U-test.

(D) A comparison of affinity of antibodies from plasmablasts and MBCs isolated from donor COV050 is shown (representative of $N = 2$ experiments). Bars show median; data were analyzed using a Mann-Whitney U-test.

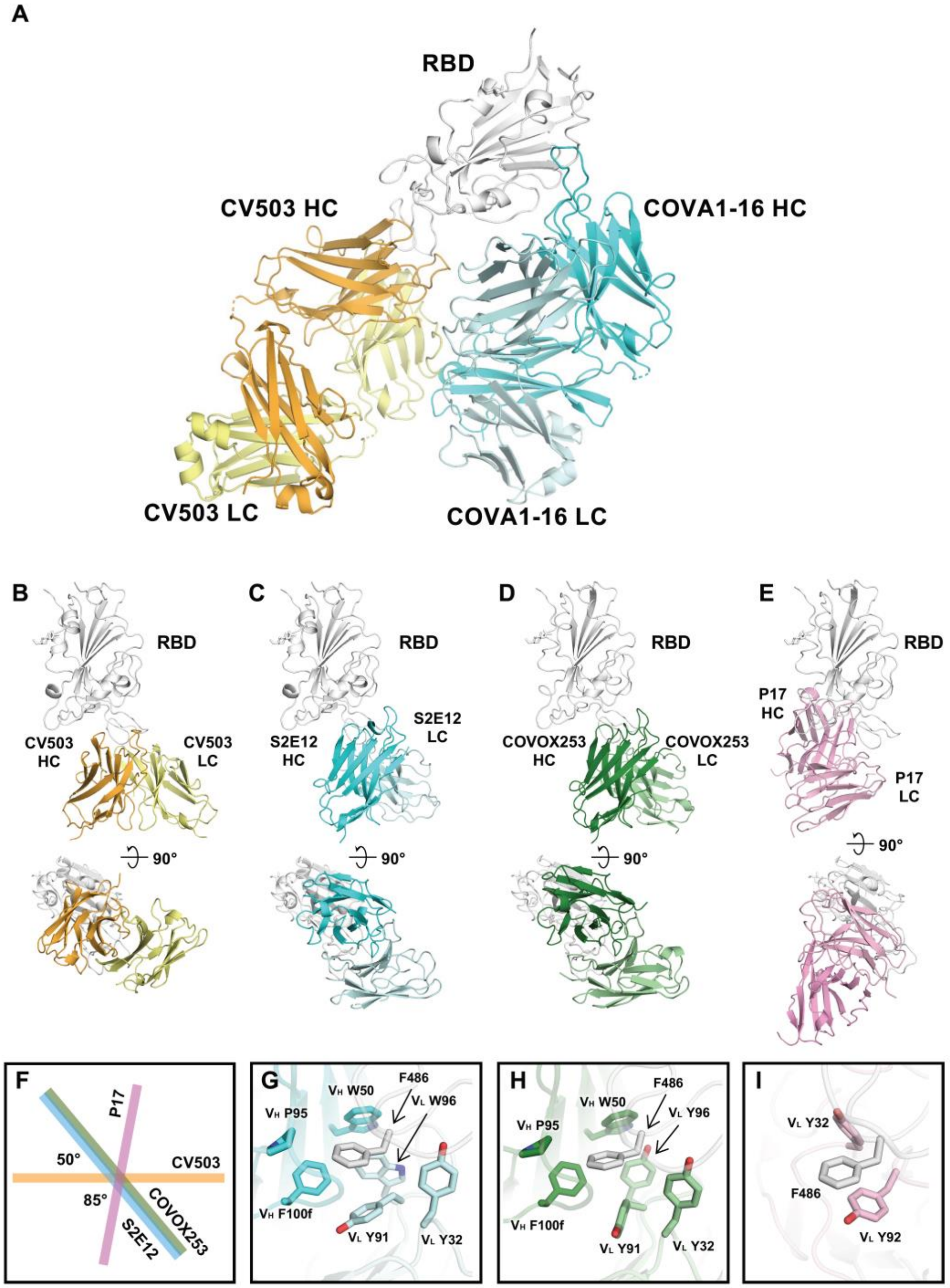


**Fig. S3. Structural comparison of CV503/RBD, S2E12/RBD, COVOX-253/RBD, and P17/RBD complexes.**
(A) Crystal structure of SARS-CoV-2 RBD in complex with Fabs CV503 and COVA1-16 is shown. The binding site of CV503 with the Fab heavy (HC) and light (LC) chains shown in orange and yellow, respectively, on the RBD (white). The epitope is distinct from that of COVA1-16, whose Fab heavy and light chains are shown in cyan and pale cyan, respectively.
(B to E) Structures of CV503/RBD, S2E12/RBD (PDB ID: 7K45, *(21)*), COVOX-253/RBD (PDB ID: 7BEN, *(36)*), and P17/RBD (PDB ID: 7CWO, *(35)*) complexes are shown, where the RBD molecules (white) are shown in the same view. Only the variable domains of the Fabs are shown for clarity.

(F) S2E12 (cyan) and COVOX-253 (green) bind to RBD in a nearly identical approach, which are rotated by 50° compared to CV503 (orange), whereas P17 (pink) is rotated by 85° compared to CV503. The rotation angles are represented by insert.
(G and H) F486 of SARS-CoV-2 RBD (white) is clamped in a hydrophobic pocket formed by six aromatic residues from the heavy (cyan) and light chains (light cyan) of S2E12 (G) or from the heavy (dark green) and light chains (light green) of COVOX253 (H).
(I) RBD-F486 stacks with $V_L$ 32 and $V_L$ 92 of P17 (pink) are shown. Kabat numbering is assigned to the antibody residues.

**A**

Dose 1 (2x $IC_{50}$)

| | Buffer | CV503 | CV521 | CV664 | CV993 | CV1182 |
|---|---|---|---|---|---|---|
| CV503 | 76.6 | | | | | |
| CV521 | 78.5 | 95.3 (95.0) | | | | |
| CV664 | 72.1 | 99.7 (93.5) | 92.8 (94.0) | | | |
| CV993 | 63.4 | 84.1 (91.4) | 95.0 (92.1) | 97.9 (89.8) | | |
| CV1182 | 69.7 | | 94.4 (93.5) | 81.9 (91.5) | 87.8 (88.9) | |

Dose 2 (1x $IC_{50}$)

| | Buffer | CV503 | CV521 | CV664 | CV993 | CV1182 |
|---|---|---|---|---|---|---|
| CV503 | 40.0 | | | | | |
| CV521 | 61.3 | 85.3 (76.7) | | | | |
| CV664 | 39.7 | 85.0 (63.8) | 78.1 (76.6) | | | |
| CV993 | 39.3 | 64.5 (63.6) | 79.1 (76.5) | 78.8 (63.4) | | |
| CV1182 | 36.5 | | 74.8 (75.4) | 71.0 (61.7) | 69.7 (61.5) | |

**B**

**CV503 + CV664**

**Run 1**

| | | CV664 (ng/mL) | | | | | |
|---|---|---|---|---|---|---|---|
| | | 0.0 | 19.8 | 39.6 | 79.2 | 158.3 | 316.7 |
| CV503 (ng/mL) | 0.0 | 0.0 | 15.0 | -4.9 | 23.0 | 25.3 | 45.9 |
| | 14.8 | 24.9 | 12.6 | 19.3 | 27.5 | 19.5 | 55.4 |
| | 29.6 | 14.4 | 11.2 | 27.2 | 25.1 | 47.0 | 54.7 |
| | 59.2 | 13.2 | 17.2 | 32.1 | 36.5 | 46.6 | 70.5 |
| | 118.4 | 41.9 | 36.5 | 47.6 | 68.6 | 72.1 | 91.8 |
| | 236.8 | 55.0 | 56.2 | 59.9 | 64.2 | 74.0 | 97.4 |

**Run 2**

| | | CV664 (ng/mL) | | | | | |
|---|---|---|---|---|---|---|---|
| | | 0.0 | 19.8 | 39.6 | 79.2 | 158.3 | 316.7 |
| CV503 (ng/mL) | 0.0 | 0.0 | 8.5 | 20.7 | 29.8 | 51.6 | 63.1 |
| | 14.8 | 28.9 | 25.0 | 36.0 | 36.9 | 29.7 | 84.3 |
| | 29.6 | 0.6 | 21.1 | 47.1 | 54.7 | 43.1 | 91.0 |
| | 59.2 | -2.3 | 21.8 | 61.3 | 70.2 | 80.6 | 98.1 |
| | 118.4 | 25.4 | 21.1 | 68.8 | 83.6 | 97.3 | 99.3 |
| | 236.8 | 55.2 | 68.2 | 92.9 | 92.6 | 99.1 | 99.4 |

Synergy values (Loewe's, >10 indicates synergy)
Run 1: 6.99
Run 2: 18.23

**CV664 + CV993**

**Run 1**

| | | CV664 (ng/mL) | | | | | |
|---|---|---|---|---|---|---|---|
| | | 0.0 | 19.8 | 39.6 | 79.2 | 158.3 | 316.7 |
| CV993 (ng/mL) | 0.0 | 0.0 | 15.0 | -4.9 | 23.0 | 25.3 | 45.9 |
| | 11.6 | 8.9 | 9.6 | 18.4 | 28.2 | 34.3 | 46.2 |
| | 23.2 | 15.7 | 20.5 | 25.5 | 20.3 | 18.7 | 68.8 |
| | 46.5 | 20.5 | 22.3 | 32.7 | 18.9 | 43.2 | 66.5 |
| | 92.9 | 34.5 | 32.9 | 28.9 | 30.7 | 61.5 | 66.4 |
| | 185.8 | 45.4 | 49.9 | 59.4 | 55.9 | 75.7 | 90.9 |

**Run 2**

| | | CV664 (ng/mL) | | | | | |
|---|---|---|---|---|---|---|---|
| | | 0.0 | 19.8 | 39.6 | 79.2 | 158.3 | 316.7 |
| CV993 (ng/mL) | 0.0 | 0.0 | 8.5 | 20.7 | 29.8 | 51.6 | 63.1 |
| | 11.6 | 21.0 | 14.5 | 1.6 | 35.5 | 30.2 | 90.7 |
| | 23.2 | 22.9 | 29.7 | 20.6 | 51.0 | 22.6 | 86.6 |
| | 46.5 | 13.0 | 33.8 | 37.9 | 37.8 | 53.6 | 90.5 |
| | 92.9 | 25.6 | 37.5 | 58.0 | 76.2 | 88.1 | 96.9 |
| | 185.8 | 35.5 | 44.5 | 69.1 | 74.2 | 98.3 | 99.0 |

Synergy values (Loewe's, >10 indicates synergy)
Run 1: 3.81
Run 2: 19.31

**C**

**CV503 + CV664**

| | | CV664 (ng/mL) | | | | | | | |
|---|---|---|---|---|---|---|---|---|---|
| | | 0.0 | 0.1 | 0.4 | 1.2 | 3.7 | 11.1 | 33.3 | 100.0 |
| CV503 (ng/mL) | 0.0 | 37.5 | 28.4 | -8.9 | -5.1 | 77.8 | 89.8 | 99.4 | 100.0 |
| | 0.1 | 71.4 | 23.0 | 66.6 | 8.6 | 90.5 | 72.1 | 99.6 | 100.0 |
| | 0.4 | 27.0 | 40.8 | 67.3 | 24.5 | 83.1 | 94.3 | 99.6 | 100.0 |
| | 1.2 | 67.4 | 44.4 | 45.9 | 88.5 | 73.5 | 97.8 | 100.0 | 99.1 |
| | 3.7 | 83.3 | 75.1 | 94.3 | 40.4 | 94.3 | 99.6 | 100.0 | 100.0 |
| | 11.1 | 94.7 | 98.1 | 80.2 | 98.4 | 99.8 | 100.0 | 100.0 | 100.0 |
| | 33.3 | 100.0 | 98.7 | 99.8 | 99.8 | 100.0 | 100.0 | 100.0 | 100.0 |
| | 100.0 | 100.0 | 100.0 | 100.0 | 100.0 | 100.0 | 100.0 | 100.0 | 100.0 |

**CV664 + CV993**

| | | CV993 (ng/mL) | | | | | | | |
|---|---|---|---|---|---|---|---|---|---|
| | | 0.0 | 0.1 | 0.4 | 1.2 | 3.7 | 11.1 | 33.3 | 100.0 |
| CV664 (ng/mL) | 0.0 | 48.2 | 37.1 | -11.2 | 37.2 | -11.4 | 86.4 | 68.3 | 98.7 |
| | 0.1 | 42.7 | -20.6 | 30.1 | 40.9 | 41.3 | 79.3 | 100.0 | 99.6 |
| | 0.4 | -36.3 | 28.3 | -2.1 | 41.8 | 56.5 | 62.5 | 93.6 | 100.0 |
| | 1.2 | 37.3 | 19.8 | -12.4 | 16.8 | 44.1 | 78.8 | 100.0 | 99.7 |
| | 3.7 | 24.5 | 30.6 | 76.6 | -11.6 | 93.4 | 100.0 | 100.0 | 100.0 |
| | 11.1 | 100.0 | 96.7 | 67.2 | 97.5 | 86.5 | 100.0 | 100.0 | 100.0 |
| | 33.3 | 100.0 | 98.4 | 100.0 | 100.0 | 100.0 | 100.0 | 100.0 | 100.0 |
| | 100.0 | 100.0 | 100.0 | 100.0 | 100.0 | 100.0 | 100.0 | 100.0 | 100.0 |

Synergy values (Loewe's, >10 indicates synergy)
CV503 + CV664:-3.88
CV664 + CV993: 9.08

**Fig. S4. Screening of antibody combinations for synergy in neutralizing SARS-CoV-2.**

(A) The results of a screen of antibody combinations for synergy in neutralizing authentic SARS-CoV-2 at two different antibody doses (2 x $IC_{50}$ and $IC_{50}$) is shown ($N$ = 1 experiment). Only non-overlapping pairs based on epitope binning data were tested. The numbers outside the brackets show the observed neutralization percentages and numbers in the brackets show the expected values. Combinations with an observed neutralization percentage of greater than 5% the expected value are highlighted blue.

(B) Neutralization of authentic SARS-CoV-2 (FRNA assay) is shown for titrations of CV503 and CV664, as well as CV664 and CV993 ($N$ = 2 experiments shown). The numbers in the heat map show the neutralization percentages, and the numbers at the side show synergy scores for each run.

(C) Neutralization of authentic SARS-CoV-2 (Scripps assay) is shown for titrations of CV503 and CV664, as well as CV664 and CV993 ($N$ = 1 experiment). The numbers in the heat map show the neutralization percentages, and the numbers at the side show synergy scores for each run.

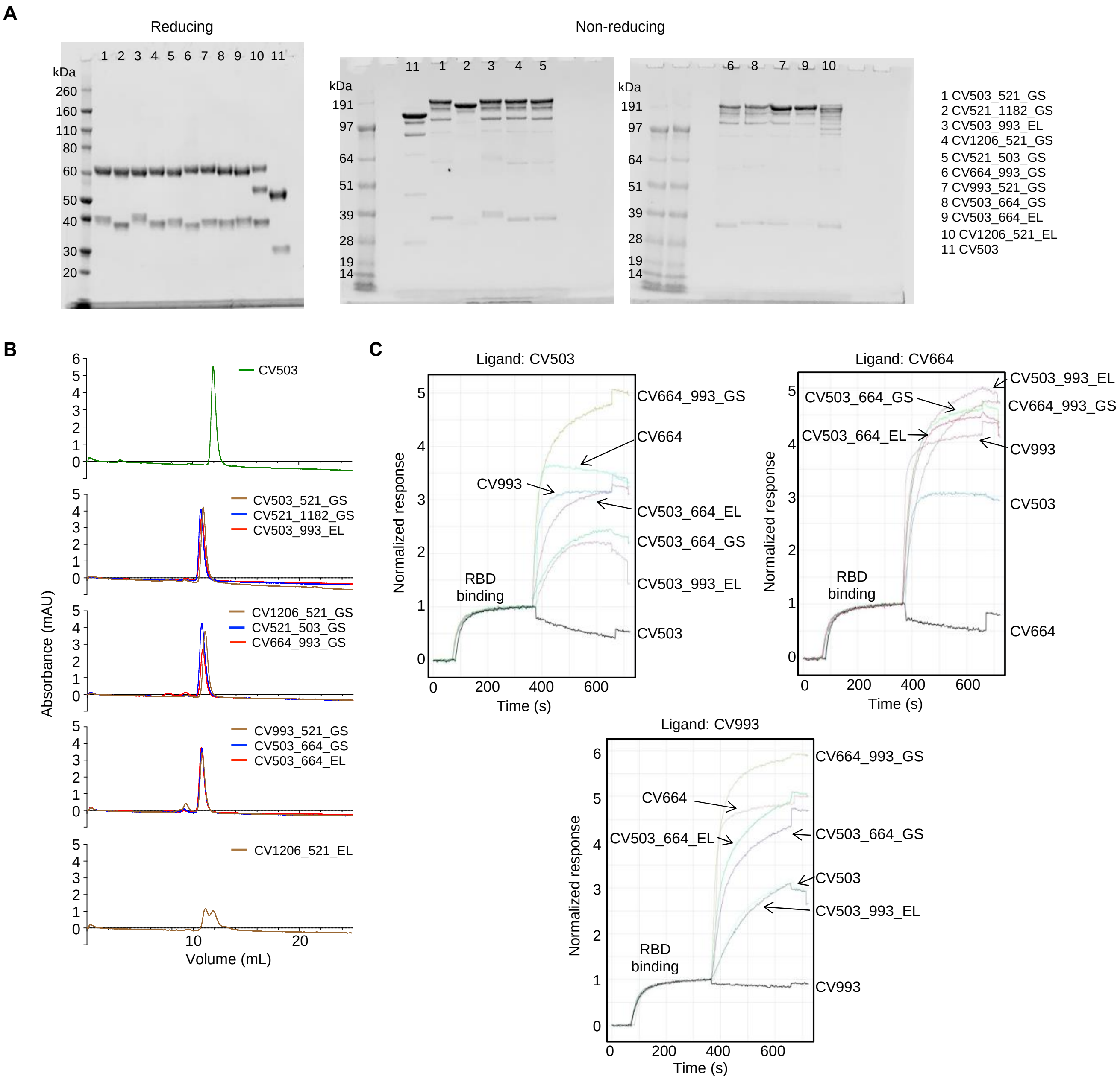


**Fig. S5. Bispecific antibodies are expressed as a single dominant species and use both binding sites.**

(A) Molecular weight of bispecific antibodies was measured by SDS-PAGE. CV503 is a control standard IgG. In our bispecific antibody naming system, the first name refers to the antibody used to make the outer binding site and the second refers to the antibody at the inner binding site. GS or EL refers to the type of linker connecting the two antigen-binding sites as described in the Materials and Methods.

(B) Size exclusion chromatography is shown for the indicated bispecific antibodies, compared to the control standard IgG CV503 ($N$ = 1 experiment).

(C) Sensograms show binding of bispecific antibodies to RBD attached to ligand IgGs (which are conjugated to the surface plasmon resonance chip). An increase in the normalized response signal after RBD binding indicates attachment of the second antibody. Representative of $N$ = 2 experiments.

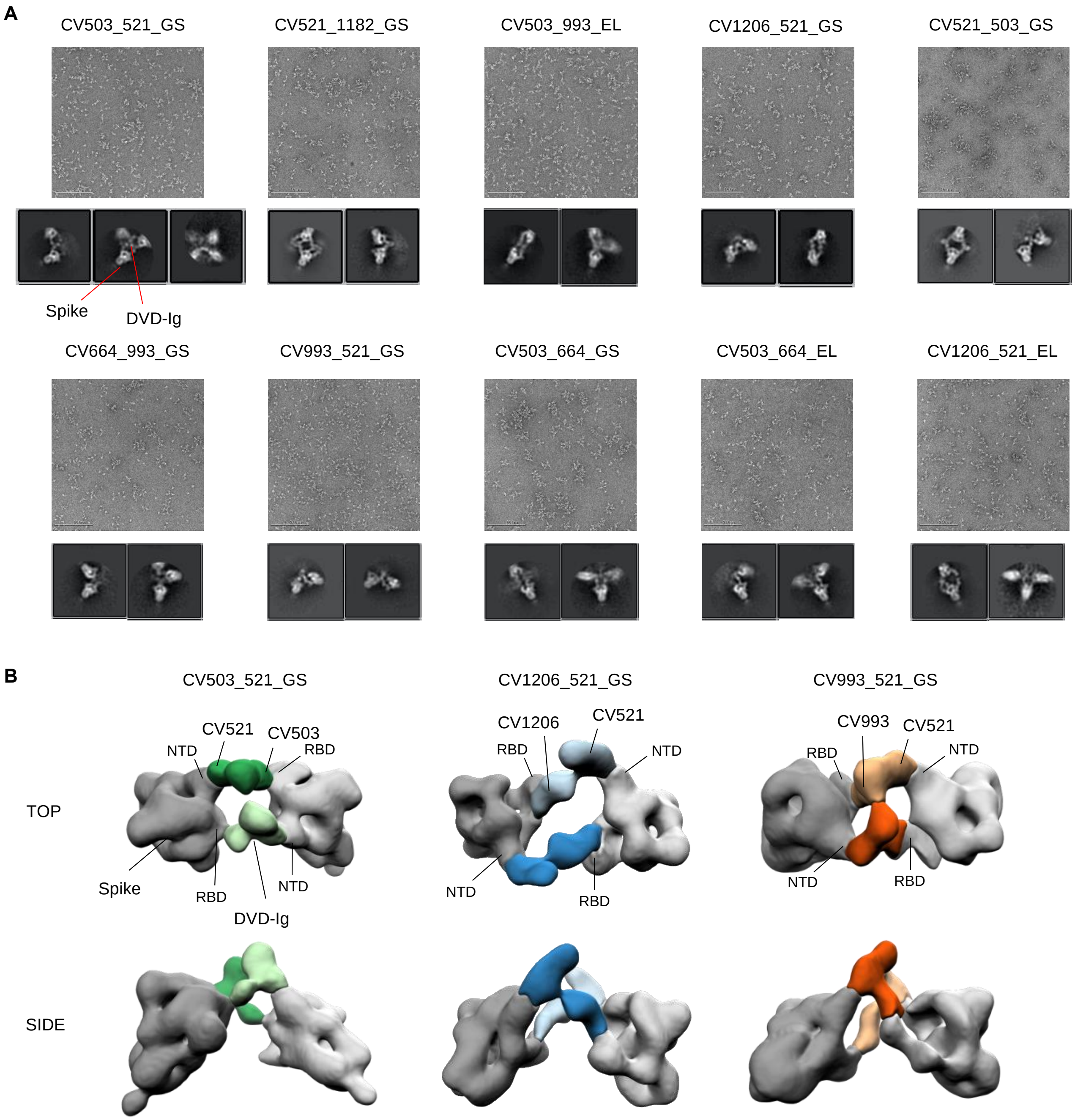


**Fig. S6. Negative stain EM images of bispecific antibodies in complex with spike protein.**

(A) Raw micrograph exemplars and select 2D classes of the bispecific antibody panel in complex with SARS-2 CoV 6P Mut7 spike protein are shown. 2D classes show the DVD-Ig induced crosslinking of 2-4 spike proteins.

(B) Segmented 3D refinements from the negative stain EM data are shown. Only 3 out of the 10 bispecific antibodies in complex with SARS-2 CoV 6P Mut7 were able to converge in 3D. Spike proteins are in gray and the bispecific antibodies are colored in green, blue, and orange for CV503_521_GS, CV1206_521_GS, and CV993_521_GS, respectively.

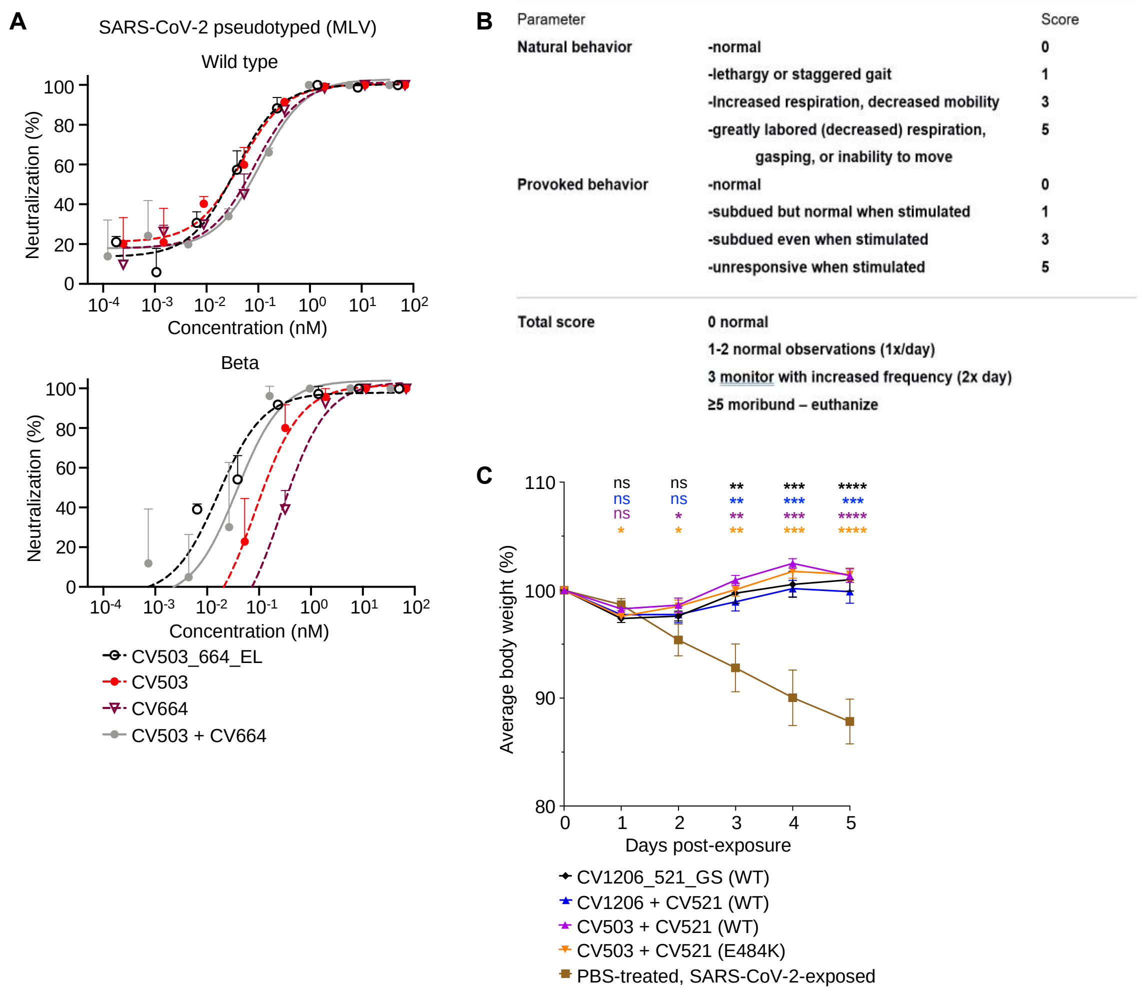


**Fig. S7. In vitro and in vivo potency of bispecific antibodies targeting SARS-CoV-2.**

(A) Potency of CV503_664_EL against wild-type and Beta SARS-CoV-2 pseudotyped virus (MLV) is shown versus individual component mAbs (representative of $N = 2$ experiments).

(B) Clinical criteria used to evaluate individual hamsters exposed to SARS-CoV-2.

(C) Weight change in hamsters that were administered bispecific antibodies at 1 mg/hamster or an equimolar mAb cocktail (0.72-0.73 mg of each mAb/hamster) 12 hours prior to intranasal virus exposure at 5 $\log_{10}$ plaque forming units (PFU). The hamsters were infected with SARS-CoV-2 USA-WA1/2020 (WT) or E484K mutant SARS-CoV-2 (E484K). Differences between groups that were given the antibody versus PBS were determined using a mixed-effects repeated measures analysis with Dunnett's multiple comparisons; *P<0.05, **P<0.01, ***P<0.001, ****P<0.0001, ns, not significant. n = 5 hamsters per group. Points show mean ± standard deviation (SD).

## Table S1. Details of mAbs isolated from plasmablasts (PB) and memory B cells (MBC)

| Name | Isotype | Donor | Cell type | K/L | VH | VL | %VH | %VL | HCDR3 | LCDR3 | COV-2 spike | COV-2 RBD | CoV-2 NTD | CoV-1 spike | CoV-1 RBD | MERS | NL63 | 229E | HKU1 | OC43 | Neut IgG (ng/mL) |
|---|---|---|---|---|---|---|---|---|---|---|---|---|---|---|---|---|---|---|---|---|---|
| CV060 | IgA2 | 5 | PB | K | 3-72 | 3-11 | 95.58 | 95.34 | 13 | 9 | 4.99 | 2.883 | -0.01 | -0.08 | -0.02 | 0.049 | 0.046 | -0.01 | 0.018 | 0.027 | >10000 |
| CV028 | IgG1 | 5 | PB | L | 3-30-3 | 3-1 | 99.31 | 99.28 | 10 | 9 | 6.14 | 0.484 | 2.078 | -0.05 | -0.02 | 0 | -0 | -0.02 | -0.02 | -0.01 | >10000 |
| CV084 | IgA1 | 5 | PB | K | 3-30-3 | 1-39 | 99.31 | 100 | 18 | 9 | 14.11 | 12.8 | 0.009 | -0.06 | 0.092 | 0.026 | -0.03 | -0.01 | 0.055 | -0 | 925.1 |
| CV067 | IgA1 | 5 | PB | L | 1-18 | 8-61 | 100 | 100 | 27 | 10 | 12 | 1.115 | -0.03 | 2.535 | -0.02 | -0.03 | 0.049 | -0.01 | -0.01 | -0.01 | >10000 |
| CV031 | IgG1 | 5 | PB | L | 1-24 | 2-14 | 100 | 99.31 | 14 | 10 | 14.1 | 0.857 | 2.911 | -0.07 | -0.03 | 0.028 | 0.039 | -0 | 0 | -0.02 | 969.3 |
| CV047 | IgG1 | 5 | PB | L | 3-9 | 2-11 | 92.36 | 92.36 | 15 | 10 | 9.57 | 5.02 | 0.049 | -0.05 | -0.03 | 0.004 | -0.02 | -0.02 | -0.05 | -0 | 5835.9 |
| CV078 | IgA1 | 5 | PB | K | 3-9 | 1-33 | 100 | 100 | 26 | 11 | 12.2 | 9.923 | -0.01 | -0.08 | -0.02 | -0.01 | 0.007 | -0.02 | -0 | -0.02 | 4048.8 |
| CV052 | IgG1 | 5 | PB | L | 3-23 | 1-40 | 99.31 | 100 | 12 | 11 | 13.37 | 1.472 | 0.025 | 2.604 | 0.019 | 0.012 | 0.044 | 0.023 | 0.025 | 0.139 | >10000 |
| CV037 | IgG1 | 5 | PB | L | 3-30 | 2-11 | 100 | 100 | 15 | 10 | 12.9 | 9.509 | -0 | 0.044 | -0.01 | 0.019 | 0.011 | 0.012 | 0.157 | 0.041 | 1331.5 |
| CV039 | IgG3 | 5 | PB | L | 3-30 | 3-25 | 97.92 | 100 | 13 | 12 | 14.5 | 0.937 | 2.854 | 0.011 | 0.023 | 0.009 | 0 | -0.03 | -0.01 | -0.01 | >10000 |
| CV068 | IgA1 | 5 | PB | L | 4-4 | 2-23 | 100 | 100 | 16 | 10 | 10.59 | 0.489 | 2.849 | -0.07 | 0.012 | -0.02 | -0 | -0.03 | -0.02 | -0.03 | >10000 |
| CV049 | IgG1 | 5 | PB | L | 4-38-2 | 3-19 | 100 | 100 | 17 | 12 | 11.69 | 1.287 | 2.857 | -0.03 | 0.009 | -0.02 | 0.016 | 0.034 | -0 | -0.01 | >10000 |
| CV044 | IgG1 | 6 | PB | L | 3-33 | 3-10 | 100 | 100 | 14 | 12 | 9.34 | 0.577 | 0.086 | -0.06 | -0.02 | -0.07 | -0.04 | -0.04 | -0.06 | -0.04 | >10000 |
| CV040 | IgG1 | 6 | PB | L | 4-34 | 2-14 | 95.09 | 97.22 | 20 | 10 | 3.98 | 2.111 | -0.02 | 1.457 | -0 | 1.11 | -0.01 | 0.011 | 1.669 | 1.716 | >10000 |
| CV042 | IgG1 | 6 | PB | L | 1-46 | 6-57 | 100 | 100 | 24 | 10 | 13.73 | 1.334 | 2.669 | -0.01 | 0.058 | 0.026 | 0.037 | 0.025 | 0.018 | 0.005 | 3411.1 |
| CV064 | IgA2 | 6 | PB | L | 1-46 | 1-44 | 100 | 100 | 17 | 11 | 8.835 | 0.345 | 2.918 | -0.03 | 0 | -0.02 | 0.007 | 0.014 | 0.004 | -0 | >10000 |
| CV073 | IgA1 | 6 | PB | L | 1-46 | 3-25 | 100 | 100 | 22 | 11 | 7.69 | 0.755 | 2.854 | -0.02 | 0.016 | 0.021 | 0.002 | 0.063 | 0.037 | 0.055 | >10000 |
| CV081 | IgA1 | 6 | PB | L | 1-46 | 1-44 | 100 | 100 | 16 | 11 | 11.4 | 10.1 | -0.04 | -0.04 | 0.61 | 0.012 | 0.072 | 0.042 | 0.018 | 0.009 | 556.7 |
| CV074 | IgA1 | 6 | PB | L | 3-48 | 3-1 | 90.62 | 93.91 | 13 | 9 | 11.5 | 1.175 | 0.011 | 2.064 | -0.01 | -0.04 | -0.03 | -0.02 | -0.02 | -0.01 | >10000 |
| CV062 | IgA1 | 6 | PB | L | 5-51 | 2-14 | 94.44 | 96.53 | 11 | 11 | 5.8 | 2.48 | -0.01 | -0.09 | -0.04 | 0.004 | -0.02 | -0.03 | -0.03 | -0.04 | >10000 |
| CV077 | IgA1 | 6 | PB | K | 4-59 | 3-15 | 99.3 | 100 | 22 | 10 | 14.17 | 11.85 | 0.891 | -0.04 | 0.069 | 0.021 | 0.03 | 0.025 | 0.011 | 0.014 | 8394.4 |
| CV085 | IgA1 | 6 | PB | K | 3-66 | 3-20 | 91.93 | 92.91 | 22 | 9 | 15.34 | 13.65 | 0.083 | 0.002 | 0.208 | 0.021 | 0.058 | 0.033 | 0.009 | 0.009 | 1483.9 |
| CV043 | IgG1 | 6 | PB | K | 2-70 | 3-15 | 99.66 | 99.64 | 13 | 9 | 6.895 | 5.368 | -0.02 | -0.07 | 0.12 | 0.021 | 0.06 | 0.025 | 0.007 | 0.007 | 7553.2 |
| CV058 | IgA1 | 6 | PB | L | 2-70 | 1-40 | 99.66 | 99.31 | 14 | 11 | 8.39 | 0.651 | 2.857 | 0.026 | 0.148 | -0.01 | -0.03 | -0.07 | -0.01 | -0.02 | >10000 |
| CV030 | IgG1 | 6 | PB | K | 3-30-3 | 1-5 | 99.31 | 99.28 | 14 | 8 | 13.9 | 0.776 | 0.085 | 2.563 | 0.063 | 0.058 | 0.002 | 0.049 | 0.026 | 0.014 | >10000 |
| CV051 | IgG1 | 6 | PB | L | 1-2 | 2-23 | 98.61 | 100 | 25 | 10 | 9.426 | 6.365 | 0.035 | -0.05 | 0.003 | 0.009 | 0.026 | 0.019 | -0.01 | -0 | >10000 |
| CV086 | IgA1 | 6 | PB | L | 1-2 | 3-19 | 100 | 100 | 14 | 12 | 9.418 | 5.557 | 0.025 | 0.302 | 0.012 | 0.025 | 0.032 | 0.025 | 0.016 | -0 | >10000 |
| CV089 | IgA1 | 6 | PB | K | 1-18 | 3-15 | 100 | 100 | 27 | 11 | 14.95 | 12.42 | 0.058 | -0.07 | 0.152 | 0.067 | 0.074 | 0.058 | 0.019 | 0.037 | 2591.2 |
| CV055 | IgA1 | 6 | PB | K | 1-24 | 3-20 | 100 | 100 | 20 | 9 | 9.752 | 0.504 | 2.809 | -0.05 | -0.02 | -0.02 | 0.009 | -0.01 | -0.02 | -0.07 | >10000 |
| CV071 | IgA1 | 6 | PB | L | 1-24 | 2-8 | 99.31 | 100 | 14 | 12 | 10.52 | 0.35 | 2.798 | -0.09 | -0.06 | -0.06 | -0.04 | -0.06 | -0.07 | -0.05 | 9591.4 |
| CV032 | IgG1 | 6 | PB | L | 3-9 | 3-21 | 100 | 99.64 | 14 | 11 | 8.63 | 8.841 | 0.131 | 0.298 | 2.471 | 0.051 | 0.055 | 0.058 | 0.032 | 0.063 | >10000 |
| CV034 | IgG1 | 6 | PB | K | 3-23 | 1-5 | 100 | 100 | 13 | 8 | 5.72 | 1.106 | 0.231 | 0.044 | -0 | -0.04 | 0.007 | 0.003 | -0.02 | -0.07 | >10000 |
| CV038 | IgG1 | 6 | PB | L | 3-30 | 1-40 | 92.71 | 95.93 | 13 | 6 | 11.99 | 0.518 | 0.056 | -0.03 | -0.01 | 0.027 | 0.028 | -0.01 | -0.02 | -0.03 | >10000 |
| CV045 | IgG3 | 6 | PB | L | 3-30 | 3-25 | 97.92 | 100 | 13 | 12 | 14.1 | 0.744 | 2.818 | -0.01 | 0.033 | -0.02 | 0.007 | 0.009 | 0.016 | 0.025 | >10000 |
| CV050 | IgG1 | 6 | PB | K | 3-30 | 1-33 | 100 | 100 | 16 | 10 | 14 | 8.965 | -0.01 | 2.33 | 2.919 | -0.01 | -0.01 | 0.025 | -0.05 | -0.01 | >10000 |
| CV087 | IgA1 | 6 | PB | L | 3-30 | 3-10 | 100 | 100 | 17 | 11 | 9.975 | 0.515 | 2.858 | 0.088 | 0.003 | 0.012 | 0.03 | 0.004 | 0.002 | -0.03 | 3123.9 |
| CV093 | IgG1 | 7 | PB | L | 3-7 | 3-1 | 99.31 | 98.21 | 13 | 10 | 13.37 | 0.38 | -0.01 | 2.242 | 0.003 | -0.01 | -0.02 | 0.021 | -0.01 | -0.02 | >10000 |
| CV305 | IgG1 | 7 | MBC | K | 3-48 | 3-11 | 100 | 100 | 20 | 9 | 6.748 | 1.381 | 2.113 | -0.05 | -0.07 | -0 | -0.01 | -0.02 | -0 | -0.04 | >10000 |
| CV278 | IgG1 | 7 | MBC | K | 3-66 | 1-5 | 97.54 | 98.21 | 11 | 5 | 2.627 | 1.198 | -0.04 | -0.04 | -0.04 | -0.01 | -0.01 | -0.04 | -0.02 | -0.06 | >10000 |
| CV282 | IgG1 | 7 | MBC | K | 4-30-2 | 2-28 | 100 | 100 | 17 | 9 | 5.602 | 0.335 | 2.655 | -0.05 | -0.02 | 0.014 | -0 | -0 | -0.02 | -0.02 | >10000 |
| CV293 | IgG1 | 7 | MBC | L | 1-2 | 2-23 | 100 | 100 | 18 | 11 | 5.34 | 0.526 | 0.016 | 2.33 | -0.03 | 0.044 | -0.01 | 0.034 | 0.002 | 0.011 | >10000 |
| CV283 | IgG1 | 7 | MBC | K | 3-21 | 3-15 | 98.26 | 100 | 23 | 10 | 12.19 | 0.617 | 1.99 | -0.07 | -0.04 | 0.023 | 0.039 | -0.04 | -0.05 | 0.055 | >10000 |
| CV280 | IgG1 | 7 | MBC | K | 3-30 | 1-17 | 100 | 100 | 31 | 9 | 10.4 | 0.445 | 1.305 | -0.03 | 0.06 | -0.03 | -0.01 | 0.014 | -0.05 | 0.009 | 3805.0 |
| CV279 | IgG1 | 7 | MBC | L | 3-30 | 1-40 | 94.44 | 98.61 | 18 | 12 | 2.883 | 0.383 | 0.002 | 1.824 | -0 | -0.01 | -0.01 | -0.05 | -0.04 | -0.04 | >10000 |
| CV284 | IgG1 | 7 | MBC | K | 3-30 | 2-28 | 93.4 | 97.62 | 18 | 9 | 2.639 | 0.144 | 2.048 | 1.826 | -0.05 | 0.007 | 0.014 | 0.009 | -0.04 | -0.02 | >10000 |
| CV291 | IgG1 | 7 | MBC | K | 3-30 | 2-24 | 88.89 | 95.58 | 11 | 9 | 5.048 | 0.345 | 1.238 | 0.699 | 0.023 | -0.01 | -0.01 | 0.046 | 0.039 | 0.026 | >10000 |
| CV297 | IgA2 | 7 | MBC | K | 3-30 | 1-27 | 98.61 | 98.21 | 19 | 9 | 7.476 | 5.733 | -0.01 | -0.06 | -0.02 | 0.018 | 0 | 0.004 | -0 | -0.02 | >10000 |
| CV299 | IgA1 | 7 | MBC | L | 3-30 | 3-21 | 95.49 | 96.77 | 14 | 12 | 3.817 | 0.421 | 0.018 | 0.314 | -0.01 | -0.02 | -0.01 | -0.01 | -0.05 | -0.04 | >10000 |
| CV657 | IgG1 | 26 | MBC | K | 3-53 | 3-20 | 98.6 | 97.87 | 11 | 9 | 13.61 | 12.14 | 0.337 | -0.05 | 0.019 | 0.014 | 0.065 | 0.027 | 0.051 | 0 | 799.3 |
| CV652 | IgG1 | 26 | MBC | L | 1-58 | 3-1 | 98.26 | 98.21 | 18 | 8 | 14.69 | 13.19 | 0.037 | 2.18 | 2.931 | 0.04 | 0.019 | 0.016 | 0.026 | -0.02 | >10000 |
| CV661 | IgG1 | 26 | MBC | L | 1-69 | 2-14 | 98.96 | 97.57 | 22 | 10 | 14.97 | 13.69 | 0.032 | 0.476 | 0.136 | -0.03 | 0.016 | -0.01 | -0.04 | -0.04 | 863.2 |
| CV664 | IgG1 | 26 | MBC | K | 3-21 | 3-11 | 96.53 | 98.57 | 16 | 9 | 16.11 | 15.53 | 0.005 | -0.05 | 0.601 | -0.01 | 0.009 | 0.011 | 0.016 | -0.02 | 78.1 |
| CV935 | IgG1 | 41 | PB | K | 4-31 | 3-11 | 100 | 100 | 14 | 11 | 8.056 | 0.905 | -0.02 | 2.44 | -0.02 | 0 | -0.05 | -0.05 | -0.02 | -0.05 | >10000 |
| CV969 | IgG1 | 41 | PB | K | 4-39 | 1-5 | 99.66 | 99.64 | 17 | 11 | 11.19 | 6.849 | -0 | 0.868 | 2.328 | -0.01 | 0.016 | -0.04 | -0.05 | 0.009 | >10000 |
| CV958 | IgG1 | 41 | PB | L | 1-2 | 2-14 | 100 | 100 | 18 | 10 | 12.77 | 10.46 | -0.01 | -0.06 | 0.005 | 0 | 0.044 | 0.011 | 0 | 0.039 | 3206.1 |
| CV972 | IgA2 | 41 | PB | L | 3-30 | 3-19 | 92.71 | 98.92 | 13 | 13 | 2.594 | 1.169 | 0 | 0.34 | -0.02 | -0.02 | -0.03 | -0.04 | -0.07 | -0.04 | >10000 |
| CV977 | IgG1 | 41 | PB | K | 3-30 | 3-11 | 100 | 98.92 | 16 | 9 | 4.613 | 1.103 | 0.15 | 1.349 | 1.087 | 0.011 | 0 | 0.041 | 0.002 | -0.02 | >10000 |
| CV990 | IgG1 | 41 | PB | K | 3-30 | 1-5 | 100 | 99.64 | 25 | 9 | 13.87 | 1.381 | 2.682 | -0.08 | 0.011 | 0.003 | -0 | -0.02 | 0 | 0.012 | >10000 |
| CV1060 | IgG1 | 47 | PB | L | 3-33 | 1-44 | 100 | 100 | 22 | 11 | 7.777 | 4.585 | -0.02 | 1.032 | 2.205 | -0.01 | -0 | -0.01 | 0.007 | -0.01 | >10000 |
| CV1071 | IgG1 | 47 | PB | L | 3-33 | 3-10 | 99.31 | 100 | 14 | 11 | 9.759 | 0.898 | 0.062 | -0.02 | 0.023 | 0.03 | 0.016 | 0.039 | -0.04 | 0.005 | >10000 |
| CV1136 | IgG1 | 47 | PB | L | 3-33 | 3-10 | 100 | 100 | 14 | 9 | 11.34 | 0.734 | -0.03 | 0.594 | -0.02 | 0.025 | -0.01 | -0.02 | -0.03 | -0.05 | >10000 |
| CV1106 | IgG1 | 47 | PB | L | 4-39 | 6-57 | 100 | 100 | 12 | 9 | 13.95 | 11.69 | -0.04 | 0.355 | 2.561 | -0.01 | 0.011 | 0.002 | 0.032 | -0 | 7671.8 |
| CV1134 | IgG1 | 47 | PB | K | 4-39 | 1-39 | 100 | 95.34 | 12 | 9 | 3.733 | 2.32 | 0.092 | -0.08 | 0.759 | -0.01 | 0 | 0.046 | 0.007 | 0.009 | >10000 |
| CV1153 | IgG1 | 47 | PB | L | 4-39 | 6-57 | 99.31 | 99.66 | 11 | 10 | 12.68 | 6.685 | 0.859 | -0.05 | 0.982 | -0.02 | 0.005 | 0.016 | -0.05 | -0.02 | >10000 |
| CV1105 | IgG1 | 47 | PB | K | 3-43 | 1-33 | 100 | 98.92 | 17 | 8 | 11.52 | 0.446 | 2.715 | -0.07 | 0.051 | -0.04 | -0.05 | -0.02 | -0.04 | -0.02 | >10000 |
| CV1097 | IgG1 | 47 | PB | L | 1-46 | 1-44 | 100 | 100 | 16 | 11 | 11.52 | 9.952 | -0.04 | -0.08 | -0.01 | -0.03 | 0.014 | -0.01 | 0.002 | -0.04 | 3447.9 |
| CV1159 | IgG1 | 47 | PB | K | 1-46 | 1-39 | 99.65 | 99.64 | 9 | 9 | 4.736 | 0.637 | -0.02 | -0.08 | -0.02 | -0.04 | 0.048 | -0.01 | -0.01 | -0.04 | >10000 |
| CV1165 | IgG1 | 47 | PB | L | 1-46 | 1-40 | 99.65 | 99.65 | 19 | 11 | 13.8 | 6.999 | -0.02 | 2.235 | 2.77 | 0.021 | -0.03 | 0.04 | 0.019 | 0.049 | >10000 |
| CV1172 | IgG3 | 47 | PB | L | 1-46 | 1-47 | 100 | 100 | 19 | 11 | 2.817 | 0.84 | -0.02 | 1.288 | -0.04 | 0.035 | 0.002 | 0.026 | 0.016 | -0.01 | >10000 |
| CV1024 | IgG2 | 47 | PB | K | 3-53 | 1-39 | 91.93 | 99.64 | 15 | 9 | 2.139 | 1.147 | -0.03 | 0.617 | -0.03 | -0.03 | -0.05 | -0.04 | -0.02 | -0.02 | >10000 |
| CV1058 | IgG1 | 47 | PB | K | 3-53 | 1-9 | 99.3 | 100 | 11 | 9 | 13.2 | 11.45 | -0.04 | -0.08 | 0.148 | 0.028 | 0.03 | 0.039 | -0 | 0.023 | 540.1 |
| CV1049 | IgG2 | 47 | PB | L | 4-59 | 7-43 | 99.65 | 99.31 | 12 | 9 | 3.095 | 1.091 | 0.968 | -0.08 | 0.034 | 0.03 | 0.009 | -0.02 | -0.04 | -0.04 | >10000 |

Binding to COV-2 spike and RBD is reported as AUC as titrations were performed.

Binding to other antigens is reported as MFI (single concentration tested).

## Table S1 (continued). Details of mAbs isolated from plasmablasts (PB) and memory B cells (MBC)

| Name | Isotype | Donor | Cell type | K/L | VH | VL | %VH | %VL | HCDR3 | LCDR3 | COV-2 spike | COV-2 RBD | CoV-2 NTD | CoV-1 spike | CoV-1 RBD | MERS | NL63 | 229E | HKU1 | OC43 | Neut IgG (ng/mL) |
|---|---|---|---|---|---|---|---|---|---|---|---|---|---|---|---|---|---|---|---|---|---|
| CV1149 | IgG1 | 47 | PB | K | 4-59 | 1-5 | 98.6 | 98.57 | 19 | 10 | 8.906 | 0.914 | -0.03 | -0.07 | -0.02 | 0.016 | 0.051 | 0.03 | 0.021 | 0.037 | >10000 |
| CV1166 | IgG1 | 47 | PB | L | 4-59 | 2-11 | 91.93 | 100 | 15 | 11 | 3.872 | 0.66 | 1.87 | -0.04 | -0.02 | 0.023 | 0.007 | 0.005 | 0.009 | -0.02 | >10000 |
| CV1176 | IgG2 | 47 | PB | L | 4-59 | 6-57 | 99.65 | 100 | 12 | 9 | 12.57 | 9.881 | -0.06 | -0.06 | 1.921 | 0.06 | 0.046 | 0.055 | 0.062 | 0.03 | >10000 |
| CV1025 | IgG1 | 47 | PB | L | 4-61 | 6-57 | 98.97 | 99.66 | 12 | 9 | 13.31 | 10.33 | -0.02 | -0.08 | 0.953 | -0.02 | 0.004 | 0.028 | -0.01 | -0.02 | >10000 |
| CV1117 | IgG1 | 47 | PB | K | 4-61 | 1-12 | 99.66 | 99.64 | 12 | 9 | 2.259 | 0.95 | 0.023 | -0.04 | 0.048 | 0.011 | -0.02 | 0.009 | 0.023 | -0.01 | >10000 |
| CV1121 | IgA1 | 47 | PB | K | 4-61 | 3-15 | 95.88 | 98.21 | 18 | 9 | 9.21 | 0.526 | 2.733 | -0.06 | -0.01 | -0.04 | -0.02 | 0.03 | -0.02 | -0.03 | >10000 |
| CV1052 | IgA1 | 47 | PB | L | 3-66 | 2-8 | 85.96 | 95.14 | 17 | 10 | 4.05 | 1.135 | 0.212 | -0.07 | 0.083 | -0.01 | 0.016 | -0.04 | 0.004 | -0 | >10000 |
| CV1018 | IgG1 | 47 | PB | L | 1-69 | 3-19 | 99.31 | 99.64 | 10 | 11 | 3.335 | 1.514 | 0.081 | -0.04 | 0.169 | -0.03 | -0.03 | -0.02 | -0 | -0.02 | >10000 |
| CV1043 | IgG1 | 47 | PB | L | 1-69 | 2-18 | 99.65 | 91.32 | 15 | 10 | 5.28 | 1.873 | 0.337 | -0.04 | 0.489 | 0.101 | 0.048 | 0.065 | 0.122 | 0.041 | >10000 |
| CV1063 | IgG3 | 47 | PB | L | 1-69 | 1-47 | 99.31 | 100 | 18 | 12 | 14.68 | 12.4 | -0.01 | -0.03 | -0.01 | 0.03 | 0.021 | 0.025 | 0.046 | 0.011 | 9313.2 |
| CV1182 | IgG1 | 47 | PB | K | 1-69 | 3-20 | 98.96 | 100 | 18 | 9 | 16.21 | 15.57 | 0.019 | -0.06 | 0.34 | 0.021 | -0.05 | 0.005 | -0.03 | 0.005 | 89.4 |
| CV1011 | IgG1 | 47 | PB | K | 2-70 | 1-39 | 99.66 | 98.92 | 12 | 9 | 5.18 | 3.83 | 0.173 | -0.04 | 0.168 | 0.018 | -0.01 | 0.018 | 0.004 | -0.01 | >10000 |
| CV1184 | IgG1 | 47 | PB | K | 2-70 | 1-39 | 100 | 98.92 | 11 | 9 | 3.216 | 1.584 | 0.016 | 0.554 | 0.208 | -0.02 | -0.02 | -0.01 | -0 | -0.01 | >10000 |
| CV1017 | IgA1 | 47 | PB | K | 1-2 | 1-39 | 96.88 | 95.7 | 12 | 9 | 7.34 | 0.467 | 2.78 | -0.05 | 0.021 | 0.023 | 0.012 | 0.028 | 0.011 | -0 | >10000 |
| CV1089 | IgG1 | 47 | PB | L | 1-2 | 2-23 | 100 | 100 | 17 | 10 | 9.425 | 0.676 | 2.765 | -0.07 | 0.021 | -0.02 | 0.021 | -0.02 | -0.01 | -0.03 | >10000 |
| CV1125 | IgG1 | 47 | PB | L | 1-2 | 3-25 | 99.65 | 99.64 | 6 | 11 | 13.82 | 0.531 | 1.217 | -0.08 | -0.01 | -0.05 | -0.02 | -0.02 | -0.02 | -0.02 | >10000 |
| CV1141 | IgM | 47 | PB | L | 1-2 | 3-21 | 100 | 100 | 16 | 12 | 11.66 | 9.203 | -0.03 | -0.08 | 0.004 | 0.004 | -0.02 | 0.025 | -0.01 | -0.02 | >10000 |
| CV999 | IgG3 | 47 | PB | K | 1-18 | 4-1 | 100 | 100 | 20 | 9 | 9.515 | 1.454 | 2.562 | -0.02 | 0.238 | 0.004 | 0.025 | 0.021 | 0.002 | 0.002 | >10000 |
| CV1016 | IgG1 | 47 | PB | L | 1-18 | 1-40 | 100 | 99.65 | 21 | 11 | 13.17 | 10.24 | 0.362 | 1.469 | 2.703 | 0.011 | 0.03 | -0.01 | 0 | 0.019 | >10000 |
| CV1036 | IgG1 | 47 | PB | L | 1-18 | 3-25 | 98.96 | 96.42 | 15 | 10 | 2.475 | 1.327 | 0.064 | -0.04 | 0.018 | 0.007 | -0.06 | 0.002 | -0.02 | -0.03 | ND |
| CV1050 | IgG1 | 47 | PB | K | 1-18 | 6D-21 | 100 | 100 | 13 | 9 | 9.064 | 9.136 | -0.01 | 2.023 | 2.924 | -0 | 0.025 | 0.02 | -0.01 | 0 | >10000 |
| CV1098 | IgG1 | 47 | PB | L | 1-18 | 3-27 | 98.61 | 100 | 13 | 11 | 13.96 | 0.647 | 2.904 | -0.07 | 0.025 | -0.02 | 0.023 | -0.02 | -0.05 | -0.01 | >10000 |
| CV1030 | IgG1 | 47 | PB | K | 3-7 | 1-12 | 99.65 | 99.64 | 18 | 9 | 2.471 | 0.202 | 0.072 | -0.08 | 0.007 | 0 | -0.04 | -0.04 | 0.018 | -0 | >10000 |
| CV1076 | IgG1 | 47 | PB | K | 3-7 | 1-5 | 98.61 | 100 | 11 | 10 | 10.9 | 8.289 | -0.02 | -0.05 | 0.959 | 0.002 | -0.03 | -0.01 | -0 | 0.002 | >10000 |
| CV1082 | IgG1 | 47 | PB | K | 3-9 | 3-20 | 98.96 | 99.29 | 16 | 10 | 12.56 | 9.898 | 0.007 | 2.372 | 2.86 | -0.01 | 0.011 | -0.03 | 0.007 | -0.02 | >10000 |
| CV1113 | IgG1 | 47 | PB | L | 3-9 | 3-25 | 100 | 100 | 14 | 11 | 3.822 | 0.576 | 0.175 | -0.06 | 0.122 | 0.048 | 0.023 | 0.042 | 0.032 | 0.044 | >10000 |
| CV1007 | IgG1 | 47 | PB | K | 3-15 | 3-20 | 100 | 100 | 26 | 7 | 14.88 | 0.511 | 2.719 | -0.06 | 0.018 | -0.03 | -0.05 | -0.03 | -0.02 | -0.01 | >10000 |
| CV1057 | IgG1 | 47 | PB | L | 3-15 | 6-57 | 99.32 | 98.63 | 14 | 9 | 10.53 | 1.024 | 2.931 | -0.03 | -0.02 | -0.01 | 0.035 | 0.028 | -0.02 | -0.04 | 1950.8 |
| CV1124 | IgG1 | 47 | PB | L | 3-15 | 6-57 | 99.66 | 99.66 | 12 | 10 | 11.44 | 9.887 | 0.012 | -0.06 | 1.549 | -0.02 | -0.02 | -0.03 | -0 | -0.04 | >10000 |
| CV1152 | IgG1 | 47 | PB | L | 3-15 | 3-1 | 99.66 | 98.92 | 14 | 8 | 13.77 | 1.277 | 2.895 | -0.05 | -0.01 | 0.04 | 0.003 | 0.003 | -0.02 | 0.002 | 1871.4 |
| CV1157 | IgG1 | 47 | PB | K | 3-15 | 1-NL1 | 99.66 | 99.64 | 24 | 9 | 15 | 13.62 | -0.04 | -0.06 | 0.688 | -0.01 | 0.026 | -0.02 | -0.01 | 0.002 | 649.8 |
| CV1026 | IgG1 | 47 | PB | K | 3-21 | 3-15 | 99.65 | 99.64 | 19 | 10 | 13.6 | 1.289 | 2.549 | -0.06 | -0.04 | -0.02 | -0.03 | -0.04 | -0.03 | -0.05 | >10000 |
| CV1002 | IgG1 | 47 | PB | K | 3-30 | 1-33 | 100 | 100 | 20 | 9 | 11.07 | 6.461 | -0.03 | 1.565 | 2.835 | 0.021 | -0.03 | 0.019 | 0.032 | -0.01 | >10000 |
| CV1065 | IgG1 | 47 | PB | L | 3-30 | 3-1 | 99.65 | 100 | 10 | 9 | 6.087 | 0.897 | 2.256 | -0.01 | 0.014 | -0.04 | 0.037 | 0.018 | -0.03 | -0.02 | >10000 |
| CV1019 | IgG1 | 47 | PB | L | 4-4 | 2-23 | 100 | 99.31 | 9 | 10 | 11.35 | 1.262 | 2.886 | -0.05 | 0.009 | 0.042 | -0.01 | 0.012 | 0.027 | 0.004 | >10000 |
| CV1061 | IgG1 | 47 | PB | L | 4-4 | 2-23 | 99.65 | 100 | 13 | 10 | 10.61 | 0.823 | 2.885 | -0.06 | -0.02 | -0.08 | -0.04 | 0.002 | -0 | -0.03 | >10000 |
| CV1062 | IgG3 | 47 | PB | K | 4-4 | 3-15 | 100 | 100 | 16 | 9 | 5.762 | 0.805 | 2.297 | -0.04 | -0.04 | 0.027 | 0 | -0.02 | -0.02 | -0.02 | >10000 |
| CV1079 | IgA1 | 47 | PB | L | 4-4 | 2-23 | 99.31 | 100 | 12 | 10 | 6.134 | 0.484 | 2.335 | -0.08 | -0.07 | -0.03 | -0.04 | -0.01 | 0.021 | -0.02 | >10000 |
| CV1090 | IgG1 | 47 | PB | L | 4-4 | 2-23 | 100 | 100 | 21 | 10 | 9.208 | 0.758 | 2.52 | -0.04 | 0.205 | -0.02 | 0.055 | 0.014 | 0.032 | 0.007 | >10000 |
| CV1130 | IgG1 | 47 | PB | L | 4-4 | 2-23 | 100 | 100 | 11 | 10 | 10.49 | 0.762 | 2.797 | -0.06 | -0.01 | -0.01 | 0.009 | 0.028 | -0.01 | -0.02 | >10000 |
| CV1115 | IgG3 | 47 | PB | K | 1-69-2 | 4-1 | 100 | 99.33 | 17 | 9 | 11.18 | 0.572 | 2.677 | -0.06 | 0.108 | 0.009 | -0.04 | 0.007 | 0.016 | -0.05 | >10000 |
| CV1208 | IgG1 | 47 | MBC | K | 3-53 | 1-33 | 100 | 100 | 11 | 9 | 10.27 | 6.465 | -0.02 | -0.07 | -0.01 | -0.02 | 0.002 | 0.028 | -0.02 | -0 | >10000 |
| CV1206 | IgG1 | 47 | MBC | K | 1-69 | 2-30 | 99.31 | 100 | 12 | 9 | 15.36 | 14.47 | -0.03 | -0.06 | 0.085 | -0.01 | -0.01 | -0.01 | -0.01 | -0.01 | 479.2 |
| CV1197 | IgG1 | 49 | PB | L | 4-39 | 6-57 | 99.66 | 99.66 | 15 | 9 | 12.73 | 10.61 | 0.021 | 1.794 | 2.675 | 0.021 | 0.009 | -0.01 | -0.01 | -0.02 | >10000 |
| CV1223 | IgG1 | 49 | MBC | K | 3-13 | 1-5 | 98.6 | 100 | 19 | 11 | 12.41 | 11.15 | -0.04 | -0.08 | 0.931 | 0.009 | 0.018 | 0.016 | -0.01 | 0.007 | >10000 |
| CV607 | IgG1 | 50 | PB | L | 4-31 | 2-14 | 98.28 | 97.92 | 12 | 11 | 11.61 | 1.26 | 1.249 | 2.622 | 1.385 | 1.654 | 1.161 | 0.85 | 1.496 | 1.513 | >10000 |
| CV611 | IgG2 | 50 | PB | L | 4-34 | 7-43 | 90.53 | 92.36 | 9 | 13 | 12.64 | 3.632 | 2.807 | 2.73 | 2.835 | 2.593 | 2.319 | 1.91 | 2.48 | 2.429 | >10000 |
| CV597 | IgG1 | 50 | PB | L | 4-39 | 1-44 | 98.97 | 100 | 18 | 11 | 14.54 | 13.4 | -0.01 | -0.08 | 0.231 | -0.04 | -0.02 | 0.007 | 0.002 | 0.03 | 439.9 |
| CV622 | IgG1 | 50 | PB | L | 4-39 | 2-23 | 100 | 99.31 | 15 | 10 | 12.84 | 1.298 | 2.809 | -0.05 | -0 | 0.028 | 0.06 | 0.028 | -0 | -0.01 | >10000 |
| CV630 | IgA1 | 50 | PB | L | 4-39 | 2-14 | 99.66 | 97.57 | 13 | 10 | 14.23 | 1.241 | 2.706 | 0.078 | 0.004 | 0.051 | 0.041 | -0 | -0.01 | 0.03 | >10000 |
| CV594 | IgG1 | 50 | PB | L | 1-46 | 3-25 | 98.96 | 97.85 | 19 | 11 | 13.78 | 1.188 | 2.905 | -0.07 | 0.145 | 0.037 | 0.033 | 0.025 | 0.039 | 0.051 | 814.7 |
| CV595 | IgG1 | 50 | PB | L | 5-51 | 9-49 | 100 | 100 | 13 | 14 | 8.018 | 1.245 | -0 | -0.04 | 0.026 | -0.02 | 0.007 | -0.01 | -0.03 | -0.02 | >10000 |
| CV609 | IgG1 | 50 | PB | K | 5-51 | 1D-13 | 98.96 | 100 | 13 | 9 | 12.32 | 10.81 | -0.03 | 2.279 | 2.903 | -0.01 | 0.046 | 0.039 | 0.039 | 0.023 | >10000 |
| CV626 | IgA1 | 50 | PB | K | 3-53 | 1-39 | 99.65 | 99.28 | 16 | 10 | 11.41 | 8.661 | 0.011 | 0.153 | 0.005 | 0.007 | 0.034 | 0.023 | 0.723 | 0.616 | 6297.8 |
| CV574 | IgG1 | 50 | PB | K | 4-59 | 1-9 | 100 | 99.64 | 16 | 10 | 13.58 | 8.03 | 0.026 | 2.531 | 0.434 | 0.007 | 0.023 | 0.018 | 0 | 0.011 | >10000 |
| CV583 | IgG3 | 50 | PB | K | 4-59 | 1-5 | 90.18 | 90.94 | 10 | 10 | 12.97 | 1.387 | 1.109 | -0.07 | 0.296 | -0.02 | -0.03 | -0.01 | -0.03 | -0.03 | >10000 |
| CV635 | IgA1 | 50 | PB | L | 4-59 | 3-25 | 99.65 | 99.28 | 16 | 11 | 14.22 | 1.172 | 2.902 | 0.162 | 0.028 | 0.007 | 0.014 | 0.012 | -0.02 | -0.02 | 472.6 |
| CV576 | IgG1 | 50 | PB | K | 1-69 | 3-20 | 98.61 | 100 | 15 | 10 | 13.69 | 10.13 | -0 | 1.826 | 2.466 | -0.01 | 0.019 | 0.012 | -0.01 | 0.002 | 501.7 |
| CV602 | IgG1 | 50 | PB | K | 1-69 | 3-20 | 99.31 | 100 | 11 | 9 | 13.88 | 2.445 | 0 | 2.021 | 2.335 | 0.014 | -0.04 | 0.007 | -0.04 | -0.02 | 632.6 |
| CV639 | IgA1 | 50 | PB | K | 2-70 | 3-20 | 90.72 | 95.04 | 11 | 11 | 3.564 | 2.634 | -0.03 | -0.08 | -0.03 | -0.01 | -0.02 | -0.05 | -0.03 | -0.04 | >10000 |
| CV633 | IgA1 | 50 | PB | K | 5-10-1 | 3-20 | 98.96 | 99.29 | 18 | 9 | 9.965 | 1.205 | 1.157 | -0.08 | -0.02 | -0.04 | -0.05 | -0 | -0.04 | -0.03 | >10000 |
| CV624 | IgG2 | 50 | PB | L | 1-18 | 1-40 | 89.58 | 94.1 | 19 | 11 | 10.16 | 1.199 | 2.638 | -0.08 | 0.083 | 0.033 | -0.03 | 0.011 | -0.03 | 0 | >10000 |
| CV584 | IgG1 | 50 | PB | L | 3-23 | 1-40 | 97.22 | 98.26 | 18 | 12 | 13.33 | 1.552 | 2.766 | -0.05 | 0.076 | 0.016 | 0.021 | 0.03 | -0.01 | 0.018 | 7096.8 |
| CV603 | IgG1 | 50 | PB | K | 3-30 | 3-15 | 98.96 | 99.28 | 18 | 10 | 13.9 | 1.276 | 2.63 | 1.423 | -0.01 | 0.012 | -0.01 | -0.04 | -0.05 | -0.05 | >10000 |
| CV593 | IgG1 | 50 | PB | K | 4-4 | 3-15 | 98.96 | 98.92 | 10 | 10 | 9.45 | 1.033 | 2.631 | 0.767 | -0.05 | -0.05 | -0.05 | -0.04 | -0.02 | -0.07 | >10000 |
| CV790 | IgG1 | 50 | MBC | K | 4-31 | 1-39 | 89.35 | 91.04 | 15 | 9 | 9.492 | 3.647 | 0.035 | 0.032 | 1.713 | -0 | -0.01 | -0.04 | -0.02 | -0.05 | >10000 |
| CV789 | IgG1 | 50 | MBC | K | 4-39 | 1-39 | 99.66 | 100 | 21 | 9 | 11.13 | 9.223 | 0.002 | -0.04 | 0.053 | 0.016 | 0.037 | 0.03 | 0.041 | -0 | 2355.9 |
| CV743 | IgG3 | 50 | MBC | L | 4-39 | 1-40 | 97.25 | 97.22 | 13 | 11 | 12.1 | 0.523 | -0.01 | -0.02 | -0.03 | 0.06 | 0.067 | 0.004 | 0.063 | 0.032 | >10000 |
| CV753 | IgG1 | 50 | MBC | L | 4-39 | 2-14 | 100 | 99.31 | 19 | 10 | 11.97 | 0.512 | 0.113 | 2.581 | -0.01 | -0.04 | 0.023 | -0.04 | -0.03 | 0.004 | >10000 |
| CV784 | IgG1 | 50 | MBC | K | 4-39 | 1-17 | 99.31 | 100 | 17 | 9 | 10.36 | 0.463 | 2.745 | 0.496 | 0.009 | -0.04 | -0.05 | -0.01 | -0.06 | -0.02 | >10000 |
| CV798 | IgG1 | 50 | MBC | L | 4-39 | 3-21 | 92.78 | 94.98 | 13 | 11 | 9.17 | 5.213 | 0.243 | -0.03 | 0.009 | 0.007 | 0.007 | -0.01 | 0 | 0.004 | >10000 |
| CV755 | IgG1 | 50 | MBC | L | 3-43 | 2-14 | 100 | 99.31 | 16 | 11 | 7.891 | 0.402 | 2.862 | -0.06 | -0.05 | 0.014 | 0.009 | 0.007 | 0.012 | -0.02 | 7860.0 |
| CV774 | IgG1 | 50 | MBC | K | 3-49 | 3-15 | 90.14 | 93.55 | 17 | 8 | 12.74 | 9.927 | 0.563 | 1.341 | 0.039 | 0.012 | 0.005 | 0.007 | -0.01 | -0.01 | 6963.0 |

Binding to COV-2 spike and RBD is reported as AUC as titrations were performed.

Binding to other antigens is reported as MFI (single concentration tested).

## Table S1 (continued). Details of mAbs isolated from plasmablasts (PB) and memory B cells (MBC)

| Name | Isotype | Donor | Cell type | K/L | VH | VL | %VH | %VL | HCDR3 | LCDR3 | COV-2 spike | COV-2 RBD | CoV-2 NTD | CoV-1 spike | CoV-1 RBD | MERS | NL63 | 229E | HKU1 | OC43 | Neut IgG (ng/mL) |
|---|---|---|---|---|---|---|---|---|---|---|---|---|---|---|---|---|---|---|---|---|---|
| CV779 | IgG1 | 50 | MBC | K | 3-53 | 1-12 | 100 | 99.64 | 11 | 10 | 13.63 | 11.73 | -0.02 | -0.06 | -0.01 | 0.007 | -0.02 | 0.011 | -0 | -0.02 | 1509.9 |
| CV772 | IgG1 | 50 | MBC | L | 4-59 | 3-1 | 100 | 100 | 14 | 8 | 4.825 | 0.74 | 2.737 | -0.03 | 0.011 | 0.014 | -0.02 | 0.012 | 0.03 | 0.027 | >10000 |
| CV752 | IgG1 | 50 | MBC | L | 4-59 | 1-44 | 100 | 100 | 11 | 11 | 4.639 | 0.499 | 2.069 | -0.06 | 0 | 0.078 | 0.041 | 0.041 | 0.053 | 0.064 | >10000 |
| CV807 | IgG1 | 50 | MBC | L | 4-59 | 3-9 | 97.54 | 97.85 | 22 | 8 | 12.22 | 9.765 | 0.564 | -0.07 | 0.088 | 0.011 | 0.023 | -0.01 | 0.002 | 0.012 | >10000 |
| CV762 | IgG1 | 50 | MBC | K | 1-69 | 3-20 | 99.31 | 98.58 | 21 | 11 | 13.1 | 9.963 | 1.266 | 2.404 | 2.881 | -0.05 | 0.009 | 0.016 | -0.03 | -0.02 | >10000 |
| CV766 | IgG1 | 50 | MBC | K | 1-69 | 3D-20 | 100 | 100 | 21 | 9 | 3.447 | 1.886 | 0.033 | -0.04 | 0.021 | 0.063 | -0.02 | 0.005 | 0.012 | -0.02 | >10000 |
| CV778 | IgG1 | 50 | MBC | L | 1-24 | 2-14 | 100 | 99.31 | 14 | 10 | 12.18 | 0.564 | 2.884 | 0.859 | -0.03 | -0.02 | 0.023 | -0.04 | -0.03 | -0.01 | 8365.4 |
| CV760 | IgG1 | 50 | MBC | L | 2-5 | 1-47 | 100 | 98.6 | 14 | 11 | 8.497 | 0.942 | 2.791 | -0.04 | 0.051 | -0.01 | -0.03 | -0.03 | -0.02 | -0.01 | >10000 |
| CV731 | IgG2 | 50 | MBC | L | 3-7 | 7-46 | 93.75 | 95.14 | 8 | 11 | 8.415 | 5.63 | -0.05 | -0.06 | -0.02 | 0.018 | 0.023 | -0.01 | 0.032 | 0.005 | >10000 |
| CV739 | IgG1 | 50 | MBC | L | 3-7 | 7-46 | 96.88 | 96.53 | 8 | 11 | 8.429 | 5.76 | 0.025 | -0.04 | -0.01 | 0.007 | 0.009 | 0 | 0.018 | -0.01 | >10000 |
| CV820 | IgG1 | 50 | MBC | K | 3-15 | 2-28 | 99.66 | 100 | 24 | 9 | 13.07 | 11.73 | 0.009 | -0.05 | 0.025 | 0.005 | 0.046 | 0.046 | 0.058 | 0.057 | 759.4 |
| CV746 | IgG1 | 50 | MBC | K | 3-15 | 1-5 | 98.98 | 99.28 | 9 | 9 | 12.89 | 0.537 | -0.05 | 2.602 | -0.04 | -0.01 | -0.01 | -0.01 | 0.011 | -0.02 | >10000 |
| CV761 | IgG1 | 50 | MBC | L | 3-23 | 3-25 | 89.93 | 93.91 | 13 | 11 | 5.015 | 0.66 | -0.02 | -0.09 | -0.04 | -0.03 | -0.04 | -0.03 | -0.02 | -0.06 | >10000 |
| CV757 | IgG1 | 50 | MBC | L | 3-30 | 1-51 | 91.32 | 95.09 | 26 | 11 | 10.63 | 0.511 | 2.681 | -0.06 | -0.01 | 0.012 | 0.014 | 0.004 | 0.016 | 0.012 | >10000 |
| CV782 | IgG1 | 50 | MBC | L | 3-30 | 1-44 | 95.49 | 95.44 | 18 | 11 | 4.285 | 0.757 | 0.383 | 2.104 | 0.758 | 0.009 | 0.051 | 0.048 | 1.917 | 1.94 | >10000 |
| CV796 | IgG1 | 50 | MBC | L | 4-4 | 2-14 | 99.31 | 98.96 | 13 | 10 | 11.31 | 9.059 | -0 | -0.08 | 0.053 | -0.01 | -0.05 | -0.04 | -0.03 | -0.02 | 2925.0 |
| CV759 | IgG1 | 50 | MBC | L | 4-4 | 2-23 | 100 | 100 | 8 | 10 | 10.87 | 0.558 | 2.816 | -0.05 | -0.06 | -0.01 | -0.03 | -0.04 | -0.02 | -0.04 | >10000 |
| CV936 | IgG1 | 55 | PB | L | 3-33 | 3-10 | 98.96 | 98.92 | 14 | 11 | 10.99 | 0.935 | 0.026 | -0.03 | 0.021 | 0.012 | -0.05 | -0.02 | -0.02 | -0.02 | >10000 |
| CV981 | IgG1 | 55 | PB | L | 3-33 | 3-10 | 99.31 | 100 | 14 | 11 | 11.25 | 1.086 | 0 | -0.06 | -0.03 | 0.009 | -0.08 | -0.05 | -0.03 | -0.01 | >10000 |
| CV963 | IgG1 | 55 | PB | L | 4-39 | 2-23 | 95.53 | 97.57 | 15 | 10 | 6.607 | 5.504 | 0.023 | -0.07 | 0.868 | 0.025 | -0.03 | -0.01 | -0.02 | -0.03 | >10000 |
| CV941 | IgG1 | 55 | PB | L | 3-53 | 3-21 | 97.19 | 99.64 | 12 | 11 | 11.48 | 9.716 | -0.02 | -0.07 | 0.018 | 0.021 | 0.025 | 0.018 | 0.011 | -0.01 | 1084.8 |
| CV525 | IgG1 | 55 | PB | K | 4-59 | 3-20 | 97.54 | 99.65 | 6 | 9 | 10.97 | 0.263 | -0.03 | 2.598 | 0.004 | -0 | -0.01 | 0.005 | 0 | -0.02 | >10000 |
| CV532 | IgG1 | 55 | PB | L | 4-59 | 3-1 | 98.25 | 98.21 | 17 | 9 | 12.05 | 0.289 | 2.849 | -0.02 | -0.02 | 0.041 | 0.067 | 0.053 | 0.083 | 0.034 | 1248.1 |
| CV943 | IgG1 | 55 | PB | K | 3-66 | 1-9 | 98.6 | 99.64 | 11 | 10 | 13.76 | 12.12 | -0 | -0.06 | 0.079 | -0.05 | -0.05 | -0.02 | -0.01 | -0.06 | 674.9 |
| CV988 | IgG1 | 55 | PB | K | 3-66 | 1-5 | 97.19 | 99.28 | 10 | 9 | 12.97 | 9.305 | -0.02 | 2.284 | 2.932 | -0 | 0.016 | 0.016 | -0.01 | -0.01 | >10000 |
| CV503 | IgG1 | 55 | PB | L | 1-69 | 2-8 | 97.85 | 99.65 | 17 | 10 | 15.24 | 14.16 | -0.01 | -0.08 | 1.321 | -0 | 0.046 | 0 | 0.019 | 0.005 | 59.2 |
| CV560 | IgG1 | 55 | PB | L | 2-70 | 7-43 | 98.97 | 100 | 20 | 9 | 7.932 | 0.365 | 2.864 | -0.06 | -0.01 | -0.02 | -0.01 | -0.01 | -0.01 | -0.02 | >10000 |
| CV939 | IgG1 | 55 | PB | L | 2-70 | 7-43 | 99.66 | 99.31 | 20 | 9 | 9.238 | 0.863 | 2.887 | -0.05 | 0.046 | -0.07 | -0.02 | -0 | -0.04 | -0.07 | >10000 |
| CV975 | IgG1 | 55 | PB | L | 3-30-3 | 2-14 | 98.96 | 98.61 | 14 | 11 | 12.55 | 0.905 | 1.268 | 2.547 | 0.028 | -0.05 | 0.026 | -0.01 | 0.014 | -0.03 | >10000 |
| CV997 | IgG1 | 55 | PB | K | 3-30-3 | 1-39 | 98.26 | 100 | 14 | 9 | 13.04 | 1.331 | 0.035 | 2.531 | 0.014 | -0 | -0.06 | -0.04 | -0.01 | -0 | >10000 |
| CV944 | IgG1 | 55 | PB | K | 1-2 | 2-24 | 99.31 | 99.66 | 21 | 9 | 6.179 | 1.431 | 0.088 | 0.143 | -0.04 | 0 | 0.002 | 0.025 | -0.01 | -0.02 | >10000 |
| CV956 | IgG1 | 55 | PB | K | 1-2 | 2-40 | 98.96 | 99.66 | 14 | 9 | 14.39 | 1.483 | 2.882 | -0.06 | 0.067 | 0.004 | 0.002 | 0.028 | -0.01 | -0.01 | 616.0 |
| CV961 | IgG1 | 55 | PB | K | 1-3 | 3-15 | 99.31 | 99.64 | 18 | 10 | 6.491 | 1.243 | -0.03 | 2.189 | -0.01 | -0.03 | -0 | -0.01 | -0.01 | -0.05 | >10000 |
| CV954 | IgG1 | 55 | PB | L | 2-26 | 2-11 | 99.31 | 100 | 17 | 8 | 13.75 | 1.596 | 2.675 | 0.014 | 0.106 | -0 | 0.018 | 0 | 0.019 | -0.02 | >10000 |
| CV962 | IgG1 | 55 | PB | L | 3-7 | 3-1 | 97.57 | 99.64 | 12 | 9 | 13 | 1.375 | 0.046 | 2.591 | 0.019 | -0.01 | 0.004 | -0 | 0.014 | -0.01 | >10000 |
| CV506 | IgG1 | 55 | PB | L | 3-9 | 1-44 | 99.31 | 98.95 | 16 | 11 | 13.51 | 11.03 | -0.02 | 1.612 | 2.883 | -0.05 | 0.019 | 0.005 | 0.016 | -0.01 | 3838.5 |
| CV993 | IgG1 | 55 | PB | L | 3-9 | 3-19 | 99.65 | 98.92 | 22 | 13 | 15.4 | 14.01 | 1.492 | 2.473 | 2.911 | -0.01 | 0.03 | -0.04 | -0.01 | -0.02 | 46.5 |
| CV991 | IgG1 | 55 | PB | K | 3-11 | 1-39 | 99.31 | 99.64 | 22 | 9 | 13 | 11.21 | -0.04 | 2.399 | 2.832 | 0.067 | 0.051 | 0.044 | 0.035 | 0.042 | >10000 |
| CV527 | IgG1 | 55 | PB | L | 3-15 | 2-14 | 98.64 | 93.75 | 20 | 8 | 9.234 | 0.238 | 2.506 | -0.05 | -0.01 | 0.018 | 0.019 | -0.03 | 0.019 | 0.007 | >10000 |
| CV524 | IgG3 | 55 | PB | L | 3-21 | 1-51 | 99.31 | 100 | 19 | 12 | 11.35 | 0.253 | 2.823 | -0.01 | -0.02 | -0.02 | -0.06 | -0.05 | -0.04 | -0.05 | 2715.2 |
| CV521 | IgG1 | 55 | PB | L | 3-30 | 3-10 | 97.22 | 99.64 | 19 | 11 | 12.48 | 0.339 | 2.929 | -0.08 | -0.03 | -0.01 | -0.02 | -0.05 | -0.01 | -0.04 | 133.1 |
| CV548 | IgG1 | 55 | PB | K | 3-30 | 3-20 | 98.61 | 98.94 | 14 | 10 | 11.89 | 1.9 | -0.01 | 2.554 | 0 | 0.002 | -0.09 | -0.03 | -0.05 | -0.03 | >10000 |
| CV964 | IgG1 | 55 | PB | L | 3-30 | 3-27 | 97.22 | 98.21 | 16 | 9 | 9.738 | 1.465 | 2.902 | -0.06 | -0.02 | 0.035 | 0.018 | 0.021 | -0.01 | -0.02 | 1170.6 |
| CV564 | IgG1 | 55 | PB | K | 4-4 | 3D-15 | 99.31 | 99.64 | 10 | 9 | 10.7 | 0.392 | 2.856 | 1.434 | -0.01 | -0.02 | -0.04 | 0.002 | -0.01 | -0.01 | >10000 |
| CV1213 | IgG1 | 55 | MBC | L | 1-2 | 6-57 | 99.31 | 100 | 14 | 10 | 11.85 | 8.954 | 0.033 | 1.175 | 2.057 | 0.012 | 0.044 | -0.02 | -0.03 | 0.009 | >10000 |
| CV1209 | IgG1 | 55 | MBC | K | 3-13 | 1-39 | 98.95 | 99.64 | 16 | 10 | 9.517 | 8.894 | -0.01 | 0.078 | 2.823 | 0.018 | 0.014 | 0.016 | 0.005 | 0.026 | >10000 |
| CV572 | IgA1 | 93 | PB | L | 3-33 | 3-10 | 98.61 | 99.28 | 14 | 12 | 12.35 | 1.467 | 0.257 | -0.05 | 0 | 0.019 | 0.009 | -0.02 | 0 | -0.04 | >10000 |
| CV518 | IgG1 | 93 | PB | K | 4-39 | 1D-12 | 97.94 | 99.28 | 15 | 8 | 12.87 | 0.414 | 2.856 | 2.217 | 0.069 | -0.01 | 0.033 | -0.02 | -0 | -0.02 | >10000 |
| CV565 | IgG1 | 93 | PB | K | 4-59 | 3-20 | 99.3 | 98.23 | 6 | 9 | 10.69 | 0.413 | 0.064 | 2.639 | 0 | 0.018 | 0.011 | 0.002 | -0.01 | 0.007 | >10000 |
| CV570 | IgA1 | 93 | PB | L | 3-7 | 2-14 | 99.65 | 98.96 | 11 | 12 | 5.157 | 0.997 | 0.005 | -0.03 | 0.005 | 0.003 | 0.032 | -0.02 | 0.03 | 0.005 | >10000 |
| CV573 | IgM | 93 | PB | K | 3-30 | 3-15 | 99.31 | 100 | 17 | 9 | 13.09 | 1.291 | -0.02 | 0.635 | -0.06 | 0.03 | 0.039 | 0.021 | 0.019 | 0.002 | >10000 |
| CV916 | IgA1 | 98 | PB | K | 3-53 | 3-15 | 100 | 98.92 | 11 | 10 | 11.12 | 8.068 | -0.02 | -0.05 | 0 | -0.01 | -0.02 | -0.02 | -0.02 | -0.02 | 8511.5 |
| CV883 | IgG1 | 98 | PB | K | 1-69 | 3-11 | 100 | 99.64 | 11 | 10 | 9.749 | 0.901 | 2.891 | 0.072 | -0.01 | 0.039 | 0.074 | 0.014 | 0.019 | 0.018 | 2945.4 |
| CV887 | IgG1 | 98 | PB | K | 3-74 | 3-15 | 99.31 | 98.57 | 19 | 9 | 12.04 | 1.077 | 2.777 | 1.88 | 0 | -0 | 0.016 | -0.04 | -0.03 | -0.05 | >10000 |
| CV926 | IgG1 | 98 | PB | L | 3-74 | 3-1 | 98.26 | 99.64 | 14 | 9 | 11.01 | 0.553 | -0.01 | 2.57 | -0.02 | -0.04 | -0.01 | -0.01 | -0.03 | -0.03 | >10000 |
| CV857 | IgG1 | 98 | PB | L | 3-30-3 | 3-27 | 100 | 100 | 18 | 9 | 7.302 | 1.029 | 0.508 | 1.676 | 0.039 | 0.014 | 0.002 | 0.018 | 0.009 | -0.01 | >10000 |
| CV864 | IgG1 | 98 | PB | L | 1-24 | 2-18 | 99.65 | 100 | 21 | 10 | 14.58 | 0.553 | 2.858 | -0.05 | -0.01 | -0.01 | 0.016 | 0.004 | 0.023 | -0.01 | 4363.2 |
| CV882 | IgG1 | 98 | PB | L | 2-5 | 3-1 | 99.31 | 98.57 | 12 | 10 | 14.35 | 1.269 | 0.049 | 2.554 | 0.053 | -0.02 | -0.02 | -0.05 | -0.03 | -0.04 | >10000 |
| CV902 | IgG1 | 98 | PB | K | 2-26 | 1-39 | 99.31 | 99.64 | 13 | 9 | 14.17 | 0.169 | 2.682 | -0.06 | -0 | -0.01 | 0.028 | -0.01 | -0.05 | -0.02 | >10000 |
| CV898 | IgA1 | 98 | PB | L | 3-9 | 2-23 | 98.96 | 99.65 | 21 | 12 | 12.6 | 1.289 | 2.722 | 0.236 | 1.393 | -0.02 | 0.03 | -0.03 | 0.009 | -0.02 | >10000 |
| CV859 | IgG1 | 98 | PB | K | 3-13 | 1-39 | 99.3 | 99.64 | 24 | 10 | 9.7 | 8.968 | -1.11 | -0.58 | 1.398 | -0.18 | -0.21 | -0.19 | -0.28 | -0.22 | >10000 |
| CV904 | IgG1 | 98 | PB | K | 3-23 | 1-17 | 99.31 | 100 | 10 | 10 | 8.282 | 0.758 | 2.751 | -0.04 | -0.01 | 0.063 | 0.021 | 0.011 | 0.023 | 0.002 | >10000 |
| CV877 | IgG1 | 98 | PB | K | 3-30 | 1D-12 | 100 | 99.64 | 15 | 9 | 2.433 | 1.464 | 0.31 | -0.07 | 0.543 | 0.023 | 0.018 | 0.042 | 0.065 | 0 | ND |
| CV901 | IgG1 | 98 | PB | K | 3-30 | 3-11 | 98.96 | 99.64 | 14 | 10 | 13.39 | 0.966 | -0.02 | 2.618 | 0.139 | 0.033 | 0.028 | 0.025 | 0.014 | 0.035 | >10000 |
| CV855 | IgG1 | 98 | PB | K | 6-1 | 1-5 | 99.33 | 98.92 | 15 | 9 | 13.09 | 0.85 | 2.894 | -0.07 | -0.04 | 0.041 | 0.014 | 0.025 | 0.007 | 0.03 | 1820.2 |
| CV1196 | IgG1 | 110 | PB | L | 3-48 | 3-1 | 96.18 | 98.57 | 13 | 9 | 13.61 | 0.737 | 0.021 | 2.519 | 0.005 | -0.02 | -0.01 | 0.002 | 0 | -0 | >10000 |
| CV1200 | IgG1 | 110 | PB | K | 1-69 | 1-39 | 98.96 | 99.64 | 19 | 8 | 9.564 | 8.112 | -0.03 | -0.04 | -0.02 | -0.03 | -0.01 | 0.003 | -0.02 | -0.04 | >10000 |
| CV1199 | IgG1 | 110 | PB | L | 3-30-3 | 3-1 | 97.57 | 99.28 | 13 | 10 | 14.71 | 0.591 | 0.055 | 2.351 | -0.01 | -0.06 | 0.005 | -0.03 | 0.007 | -0.01 | >10000 |
| CV1195 | IgG1 | 110 | PB | L | 2-26 | 3-25 | 98.63 | 98.92 | 14 | 9 | 11.41 | 10.1 | -0.02 | 2.096 | 2.937 | 0.005 | 0.009 | 0.023 | -0.01 | -0.01 | >10000 |
| CV1217 | IgG1 | 110 | MBC | K | 3-53 | 3-11 | 97.19 | 100 | 11 | 11 | 11.76 | 10.22 | -0.02 | -0.06 | 0.452 | 0.009 | 0.016 | 0.018 | -0.02 | -0.04 | 1692.6 |
| CV1216 | IgG1 | 110 | MBC | K | 1-69 | 1-39 | 100 | 99.64 | 17 | 8 | 15.74 | 14.87 | -0.01 | -0.01 | 0.069 | 0.413 | -0.02 | -0 | 0.003 | -0.01 | 5054.8 |

Binding to COV-2 spike and RBD is reported as AUC as titrations were performed.

Binding to other antigens is reported as MFI (single concentration tested).

**Table S2. X-ray data collection and refinement statistics.**

| **Data collection** | CV503 + RBD + COVA1-16 |
|---|---|
| Beamline | SSRL12-1 |
| Wavelength (Å) | 0.97946 |
| Space group | P 1 $2_1$ 1 |
| Unit cell parameters | |
| a, b, c (Å) | 172.2, 122.7, 175.5 |
| α, β, γ (°) | 90, 118.2, 90 |
| Resolution (Å) [a] | 50.0-3.40 (3.48-3.40) |
| Unique reflections [a] | 87,443 (8,562) |
| Redundancy [a] | 3.4 (3.5) |
| Completeness (%) [a] | 98.8 (99.8) |
| $<I/\sigma_I>$ [a] | 10.2 (1.0) |
| $R_{sym}$[b] (%) [a] | 14.6 (>100) |
| $R_{pim}$[b] (%) [a] | 4.3 (64.9) |
| $CC_{1/2}$[c] (%) [a] | 99.8 (53.6) |
| **Refinement statistics** | |
| Resolution (Å) | 40.6-3.40 |
| Reflections (work) | 87,416 |
| Reflections (test) | 2,000 |
| $R_{cryst}$[d] / $R_{free}$[e] (%) | 20.0/23.5 |
| No. of atoms | 24,307 |
| RBD | 4,671 |
| CV503 Fab | 9,602 |
| COVA1-16 Fab | 10,034 |
| Average *B*-values (Å$^2$) | 131 |
| RBD | 139 |
| CV503 Fab | 131 |
| COVA1-16 Fab | 128 |
| Wilson *B*-value (Å$^2$) | 128 |
| **Root mean square deviation (RMSD) from ideal geometry** | |
| Bond length (Å) | 0.002 |
| Bond angle (º) | 0.60 |
| **Ramachandran statistics (%)** | |
| Favored | 95.8 |
| Outliers | 0.28 |
| **PDB code** | 7LQ7 |

[a] Numbers in parentheses refer to the highest resolution shell.

[b] $R_{sym} = \Sigma_{hkl} \Sigma_i | I_{hkl,i} - <I_{hkl}> | / \Sigma_{hkl} \Sigma_i I_{hkl,i}$ and $R_{pim} = \Sigma_{hkl} (1/(n-1))^{1/2} \Sigma_i | I_{hkl,i} - <I_{hkl}> | / \Sigma_{hkl} \Sigma_i I_{hkl,i}$, where $I_{hkl,i}$ is the scaled intensity of the $i^{th}$ measurement of reflection h, k, l, $<I_{hkl}>$ is the average intensity for that reflection, and *n* is the redundancy.

[c] $CC_{1/2}$ = Pearson correlation coefficient between two random half datasets.

[d] $R_{cryst} = \Sigma_{hkl} | F_o - F_c | / \Sigma_{hkl} | F_o |$ x 100, where $F_o$ and $F_c$ are the observed and calculated structure factors, respectively.

[e] $R_{free}$ was calculated as for $R_{cryst}$, but on a test set comprising 5% of the data excluded from refinement.

**Table S3.** **Primers used to generate SARS-CoV-2 variant plasmids (MLV assay)**

| | Mutation | Forward primer | Reverse primer |
|---|---|---|---|
| B.1.1.7 | HV69-70 del | gttccatgccatctctggcaccaatggca | tggtgccagagatggcatggaaccaggtc |
| | Y144 del | cctgggagtctaccacaagaacaacaagtcct | tgttgttcttgtggtagactcccaggaatgggtca |
| | N501Y | tatggagtgggctaccaacca | ggttggtagcccactccataggttggttggaagccat |
| | A570D | gggacattgatgacaccacagatgctgtga | ctgtggtgtcatcaatgtccctgccaaact |
| | D614G | ggtgtgaactgtactgaggtgcct | acctcagtacagttcacaccctggtagagcacagccacctggt |
| | P681H | ataggagggcaaggtctgtggcaa | gccacagaccttgccctcctatggctgttggtct |
| | T716I | tcaacttcaccatctctgtgaccaca | tggtcacagagatggtgaagttgattgggatggcaatg |
| | S982A | gccagactggacaaggtggaggc | tccaccttgtccagtctggccaggatgtcattcag |
| | D1118H | cacaacacctttgtgtctggca | ccagacacaaaggtgttgtgtgtggtgataatct |
| B.1.351 | L18F | Ttcaccaccaggacccaacttcct | gaagttgggtcctggtggtgaagttcacacactggct |
| | D80A | Ccaaccctgtgctgccattcaa | gaatggcagcacagggttggcaaacctcttggtg |
| | D215G | Gcctgccacagggcttctct | gagaagccctgtggcaggcccctcaccaggtt |
| | R246I | Tctcctacctgacacctggagact | ctccaggtgtcaggtaggagatgtgcagagccagca |
| | K417N | caggcaaCattgctgactacaactacaaact | gttgtagtcagcaatgttgcctgtttgtccaggggcaa |
| | E484K | ggagtgAagggcttcaactgttactttcca | cagttgaagccctTcactccattacatggtgt |
| | N501Y | Tatggagtgggctaccaacca | ggttggtagcccactccataggttggttggaagccat |
| | D614G | gGtgtgaactgtactgaggtgcct | acctcagtacagttcacaCcctggtagagcacagccacctggt |
| | A701V | gtctgggagtagagaactctgtggcttaca | Cacagagttctctactcccagactcattgtgtag |
| P.1 | L18F | ttcaccaacaggacccaacttccttctgcctacaccaact | ggaagttgggtcctgttggtgaagttcacaca |
| | T20N | | |
| | P26S | | |
| | D138Y | gttctgtaattacccattcctgggagtctacta | tcccaggaatgggtaattacagaactggaact |
| | R190S | acctgtcggagtttgtgttcaagaacattgatg | ttgaacacaaactccgacaggttcttgaagttg |
| | K417T | CAAACAGGCACGATTGCTGACTAC | gtcagcaatcgtgcctgtttgtcca |
| | E484K | ggagtgAagggcttcaactgttactttcca | cagttgaagccctTcactccattacatggtgt |
| | N501Y | tatggagtgggctaccaacca | ggttggtagcccactccataggttggttggaagccat |
| | D614G | gGtgtgaactgtactgaggtgcct | acctcagtacagttcacaCcctggtagagcacagccacctggt |
| | H655Y | agcagagtatgtgaacaactcctatgagtgtg | ataggagttgttcacatactctgctccaatcag |
| | T1027I | tgccatcaagatgagtgagtgtgtgct | acacactcactcatcttgatggcagccaggttggcagat |
